# Targeted Metabolomics and High-Throughput RNA Sequencing-Based Transcriptomics Reveal Massive Changes in the Streptomyces venezuelae NRRL B-65442 Metabolism Caused by Ethanol Shock

**DOI:** 10.1128/spectrum.03672-22

**Published:** 2022-10-31

**Authors:** Olga N. Sekurova, Martin Zehl, Michael Predl, Peter Hunyadi, Thomas Rattei, Sergey B. Zotchev

**Affiliations:** a Department of Pharmaceutical Sciences, Division of Pharmacognosy, University of Vienna, Vienna, Austria; b Department of Analytical Chemistry, Faculty of Chemistry, University of Vienna, Vienna, Austria; c University of Vienna, Centre for Microbiology and Environmental Systems Science, Division of Computational System Biology, Vienna, Austria; d University of Vienna, Doctoral School in Microbiology and Environmental Science, Vienna, Austria; University of Michigan-Ann Arbor

**Keywords:** *Streptomyces venezuelae*, ethanol shock, metabolomics, transcriptomics

## Abstract

The species Streptomyces venezuelae is represented by several distinct strains with variable abilities to biosynthesize structurally diverse secondary metabolites. In this work, we examined the effect of ethanol shock on the transcriptome and metabolome of Streptomyces venezuelae NRRL B-65442 using high-throughput RNA sequencing (RNA-seq) and high-resolution liquid chromatography-tandem mass spectrometry (LC-MS/MS). Ethanol shock caused massive changes in the gene expression profile, differentially affecting genes for secondary metabolite biosynthesis and central metabolic pathways. Most of the data from the transcriptome analysis correlated well with the metabolome changes, including the overproduction of jadomycin congeners and a downshift in the production of desferrioxamines, legonoxamine, foroxymithin, and a small cryptic ribosomally synthesized peptide. Some of the metabolome changes, such as the overproduction of chloramphenicol, could not be explained by overexpression of the cognate biosynthetic genes but correlated with the expression profiles of genes for precursor biosynthesis. Changes in the transcriptome were also observed for several genes known to play a role in stress response in other bacteria and included at least 10 extracytoplasmic function σ factors. This study provides important new insights into the stress response in antibiotic-producing bacteria and will help to understand the complex mechanisms behind the environmental factor-induced regulation of secondary metabolite biosynthesis.

**IMPORTANCE** *Streptomyces* spp. are filamentous Gram-positive bacteria known as versatile producers of secondary metabolites, of which some have been developed into human medicines against infections and cancer. The genomes of these bacteria harbor dozens of gene clusters governing the biosynthesis of secondary metabolites (BGCs), of which most are not expressed under laboratory conditions. Detailed knowledge of the complex regulation of BGC expression is still lacking, although certain growth conditions are known to trigger the production of previously undetected secondary metabolites. In this work, we investigated the effect of ethanol shock on the production of secondary metabolites by Streptomyces venezuelae and correlated these findings with the expression of cognate BGCs and primary metabolic pathways involved in the generation of cofactors and precursors. The findings of this study set the stage for the rational manipulation of bacterial genomes aimed at enhanced production of industrially important bioactive natural products.

## INTRODUCTION

Gram-positive filamentous bacteria belonging to the genus *Streptomyces* dwell in various complex environments, such as soils, marine sediments, plants, and animals, where they are often challenged with both biotic and abiotic stress factors (1). Hence, these bacteria must be able to adjust their gene expression patterns in order to meet these challenges and to change their metabolism accordingly. One of the well-known traits of streptomycetes is their ability to biosynthesize chemically diverse secondary metabolites in response to various stress factors, such as nutrient limitation, temperature shift, and the presence of other microorganisms nearby (2). Some of these secondary metabolites have antimicrobial activity, and quite a few have been developed into medically useful antibiotics, e.g., erythromycin, daptomycin, and tetracyclines. The roles that antibiotically active secondary metabolites play in natural environments are still being debated, but mounting evidence shows that besides their involvement in the “chemical warfare” to eliminate competitors for nutrient sources (1), they also act as signaling molecules at subinhibitory concentrations (3) that can shape microbial communities (4). Understanding of the environmental factor-dependent mechanisms that trigger the biosynthesis of secondary metabolites would provide important insights into the biology of streptomycetes and may also be applied to increase the industrial yields of medically important antibiotics.

In bacteria, one- and two-component systems, as well as extracytoplasmic function σ factors (ECFs), play pivotal roles in sensing the environment and transducing the signals inside the cell, leading to specific changes in the gene transcription patterns (5). The one-component systems consist of one protein with sensing and regulatory (DNA binding) domains (6), while the two-component systems are composed of a sensor kinase and a transcriptional regulator, subject to activation by the cognate kinase via phosphorylation (7). The activity of ECFs is usually regulated by transmembrane anti-σ factors, to which they are tightly bound until their release in response to an environmental stimulus. Notably, there also exist “hybrid” systems, where ECF sigma factors contain additional sensor domains that regulate their activity along with anti-σ factors (8). Both, two-component systems and ECFs are known to control secondary metabolism in *Streptomyces* (9–11) and thus appear to be crucial for the activation of the latter in response to environmental stimuli. However, molecular details of the mechanisms behind such signal transduction remain mostly obscure due to the extreme complexity of the regulatory networks that control secondary metabolism in streptomycetes. Streptomyces venezuelae, known as a producer of the antibiotic chloramphenicol, represents an interesting study subject in this respect since it is known to respond to phage infection, temperature shift, or ethanol shock by changing its secondary metabolome (12, 13). In *S. venezuelae* ATCC 10712, ethanol stress suppresses the production of chloramphenicol while simultaneously triggering the biosynthesis of angucycline polyketides, namely, jadomycins (12), and the regulatory mechanism behind such reciprocal regulation has been studied at the level of pathway-specific regulators (14, 15).

In this work, we investigated the effect of ethanol stress on the global transcriptome of *S. venezuelae* NRRL B-65442 by means of high-throughput RNA sequencing (RNA-seq) and correlated these data with the changes in the secondary metabolome. In addition, we examined the effect of ethanol shock on the transcription of genes governing major primary metabolic pathways and genes encoding ECFs as well as proteins known to be involved in stress response. The results obtained represent a significant step toward deciphering a complex process of stress response in *Streptomyces* bacteria and its connection to secondary metabolite biosynthesis.

## RESULTS AND DISCUSSION

### Streptomyces venezuelae NRRL B-65442 genome and its BGCs and secondary metabolites.

Various strains of Streptomyces venezuelae have been investigated by several research groups (16, 17). This species has remarkable properties for streptomycetes, in that it grows very fast and sporulates almost synchronously in liquid medium. The two very closely related strains *S. venezuelae* ATCC 10712 and NRRL B-65442 received considerable attention due to their ability to activate antibiotic biosynthesis upon ethanol shock (12, 13). Gomez-Escribano et al. (16) recently compared the genomes of these strains, which differed in 46 nucleotide mismatches and 34 indels. These differences might account for the previously observed variation in the strains’ response to ethanol shock, whereby chloramphenicol biosynthesis was suppressed by it in ATCC 10712 while being stimulated in NRRL B-65442 (mistaken for ATCC 10712 in reference 13). We once again examined the genome of NRRL B-65442 using antiSMASH6.0 (18), followed by manual curation, and amended the table of BGCs proposed by Gomez-Escribano et al. (16) as shown in Table 1. Since the effect of ethanol stress on both ATCC 10712 and NRRL B-65442 has been studied in one liquid medium, namely, Maltose Yeast Extract medium (MYM) (see Materials and Methods), we decided to use the same conditions in the follow-up experiments with strain NRRL B-65442, aimed at examining its secondary metabolome.

**TABLE 1:** Secondary metabolite biosynthesis gene clusters in the genome of *S. venezuelae* NRRL B-65442 and effects of ethanol shock on their expression and cognate metabolites

BGC	Type	Known or putative product^*a*^	Gene	Metabolome	Transcriptome	Pathway
1	Ectoine	**Ectoine**/betaine	RS01090–01095	ND^*b*^	Down	P101-PWY
2	Terpene	**Geosmin**	RS01290	ND	Down	PWY-5950
3	Pks/NRPS	**Venemycin**	RS02305–02380	ND	None	None
4	NRPS	**Watasemycins/thiazostatins**	RS02405–02475	Down	None	None
5	Lantipeptide	Core: ADhaIDVPYDhbDhbGDhbIDhbVC	RS02610–02620	ND	None	None
6	Terpene	Terpenoid	RS02650–02655	ND	Down	None
7	Lantipeptide	**Venezuelin**	RS02970–02980	ND	None	None
8	Indole	Arcyriaflavin	RS03645–03670	ND	None	None
9	NRPS	**Chloramphenicol**	RS04425–04505	Up	Down/up	PWY-8032
10	CDPS	**Cyclic dipeptide**	RS09190–09195	None	None	PWY-7236
11	Siderophore	**Desferrioxamine**	RS12675–12690	Down	Down	PWY-6375
12	Lasso peptide	Core: GDAAELTQGQGGGQSEDKRRAYNC	RS15430–15450	ND	None	None
13	NRPS	Peptide	RS20250–20325	ND	None	None
	Other	**Gaburedins**	RS20785–20795	Down	Down	None
14	Butyrolactone	Butyrolactone	RS20800–20805	ND	None	None
15	Melanin	Melanin	RS23170–23175	ND	None	PWY-6481
16	Butyrolactone	Butyrolactone	RS25385	ND	None	None
17	Other	Pyrrolnitrin-like?	RS25415–25435	ND	None	PWY-6831
18	Thiopeptide	Core: SSGSIGTSSSSSSTCSAC	RS25565–25620	ND	Down	None
19	Pks3	Flaviolin	RS26780	ND	None	None
20	Siderophore	Siderophore	RS27055–27060	ND	None	None
21	Siderophore	**Legonoxamines**	RS27330–27345	Down	Down	None
22	Bacteriocin	Bacteriocin	RS29140	ND	None	None
23	Pks2	**Jadomycin**	RS29740–29955	Up	Up	PWY-6679
24	NRPS	Peptide	RS30590–30630	ND	Up	None
25	Pks2-NRPS	Peptide-polyketide hybrid	RS30820–30995	ND	Down/up	None
26	Ladderane	Ladderane	RS31000–31150	ND	Down/up	None
27	NRPS	Terpapeptide (x-Phe-X-Gly?)	RS31155–31260	ND	Down	None
28	Terpene	**Hopanoids**	RS32065–32140	ND	Down	PWY-7072
29	Bacteriocin	Bacteriocin	RS32515	ND	None	None
30	Pks2	**Spore pigment**	RS33815–33860	ND	Down	None
31	Melanin	Melanin	RS34060-34065	ND	None	None
32	NRPS	**Foroxymithines**	RS35125–35150	Down	Down	None
33	Terpene	**2-Methylisoborneol**	RS35405–35410	ND	Down	None
34	Pks3	**Alkylresorcinol**	RS36050–36065	Down	Down	None
35	Terpene	Terpenoid	RS36960	ND	None	None
36	NRPS	Peptide	RS37045	ND	Down	None

a Known secondary metabolites for which cognate BGCs are known are shown in bold.

b ND, not detected in conditions tested with the given workflow.

Previously, secondary metabolites corresponding to seven of the BGCs shown in Table 1 were reported from *S. venezuelae* ATCC 10712 and NRRL B-65442. Using a liquid chromatography-mass spectrometry (LC-MS)-based metabolomics workflow, the production of the following metabolites in MYM medium was confirmed by matching accurate mass, isotopic pattern, and tandem MS (MS/MS) data: gaburedins A to D (19); the 2-hydroxyphenylthiazolines watasemycin A/B, thiazostatin A/B, isopyochelin, pulicatin A to D, and aerugine (20); and chloramphenicol (21), as well as desferrioxamine B (22) and numerous related siderophores, such as legonoxamine A (23). The *S. venezuelae* BGC-encompassing genes RS27330 to RS27345 (Table 1) could be linked to the biosynthesis of legonaxamines, as it encoded enzymes similar to those for the biosynthesis of desferrioxamines.

Although jadomycin B could not be detected, the related angucyclines l-digitoxosyl-phenanthroviridin described for *S. venezuelae* ISP5230 (24) and, in smaller amounts, phenanthroviridin aglycone (25) were tentatively identified. l-Digitoxosyl-phenanthroviridin was proposed to be the stable degradation product of the jadomycin B analogue with l-lysine incorporated instead of l-isoleucine (24). Our data support this hypothesis since a minor peak matching this transient jadomycin l-lysine was detected in some samples. In addition, the rare siderophore foroxymithine (26) was identified in *S. venezuelae* for the first time and tentatively matched to the BGC responsible for its production (see Table 1). Venemycin (27) and venezuelin (28) were not detected, but the production of both these compounds was described only in a mutant strain or upon heterologous expression and not in the wild-type *S. venezuelae*, where the corresponding BGCs appear to be silent under these cultivation conditions.

Besides the above-mentioned natural products, a large number of other metabolites were tentatively identified, such as amino acids and their derivatives (e.g., *N*-phenylacetylglutamine), diketopiperazines [e.g., cyclo(Phe-Pro)], nucleosides (e.g., various *N*- and *O*-methyladenosines), lipids (e.g., fatty acid amides and phosphatidylethanolamines), and cofactor-related compounds (e.g., biotin and pantothenic acid).

One of the main peaks in the chromatograms of the *S. venezuelae* NRRL B-65442 culture extract turned out to be a linear ribosomal peptide of 19 amino acids long with a formyl group on the *N*-terminal methionine (Met). With lower abundance, derivatives originating from the oxidation of the Met to the sulfoxide, C-terminal truncation down to the tetrapetide, deamination of an Asn to Asp, and combinations of those were observed. The full sequence of the peptide, designated PepX, could be derived from the MS/MS spectra (with the exception of Leu and Ile, which cannot be differentiated easily in *de novo* sequencing). PepX was found to be encoded by a small gene provisionally named RS03776, located between RS03775 and RS03780 encoding a putative endolytic transglycosylase and an ABC transporter ATP-binding protein, respectively. No putative function could be assigned to the PepX peptide based on its amino acid sequence, which was translated as *N*-formyl-MNVITNLLAGVVHFLGWLV in agreement with the MS/MS data. However, the *pepX* gene homologue could be identified in genomes of various streptomycetes and appears to be conserved. High-resolution MS and MS/MS spectra of the above-mentioned compounds and more details on their identification can be found in the Supplemental Material (Fig. S1 to S9).

### Effect of the ethanol shock on the secondary metabolism of *S. venezuelae*.

**FIG 1:**
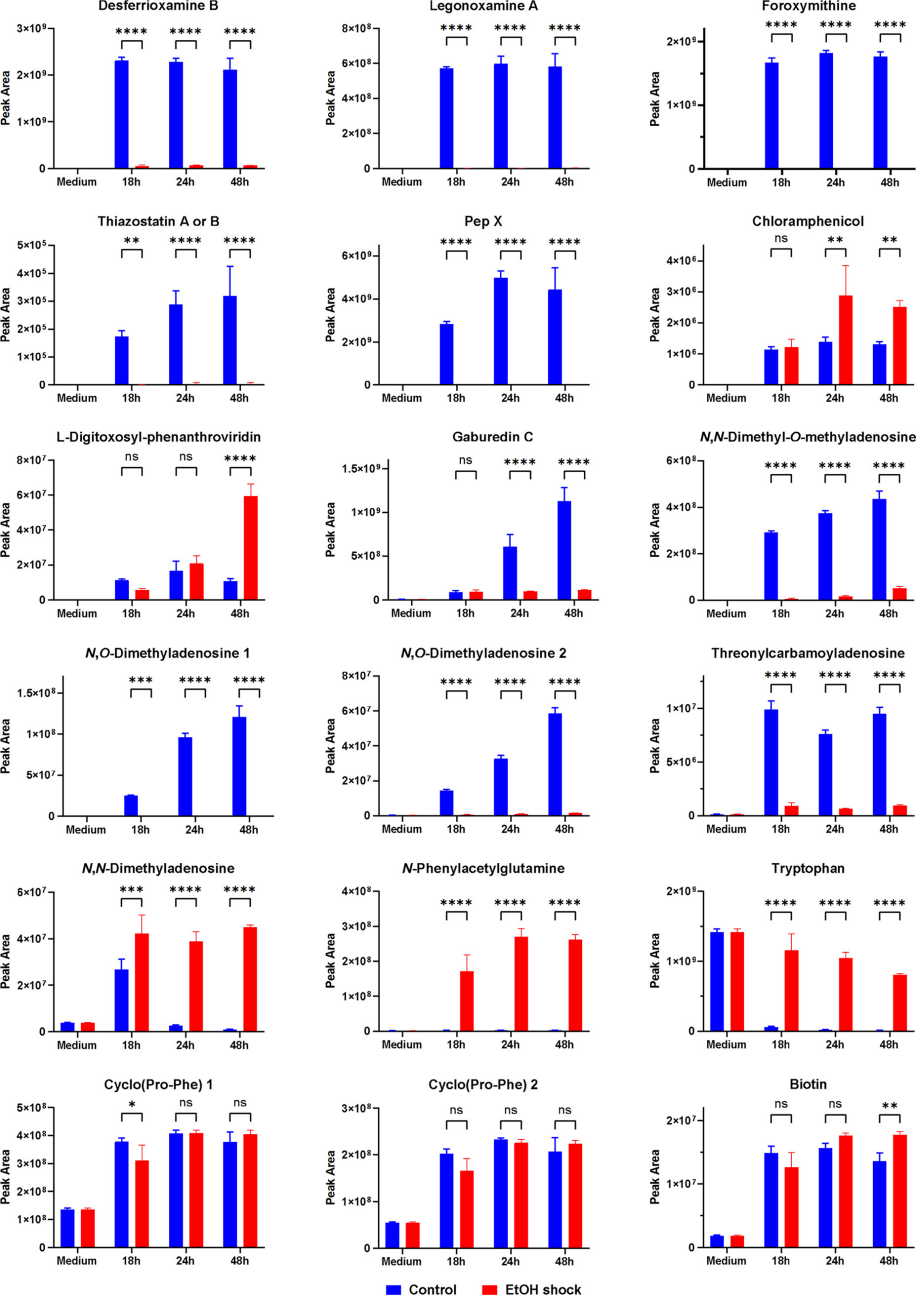
Effect of ethanol shock on the production of secondary metabolites and the relative concentration of other identified compounds in MYM cultures of *S. venezuelae* NRRL B-65442 after 18 h, 24 h, and 48 h of cultivation. After 7 h of cultivation, either 6% (vol/vol) of absolute ethanol (red bars) or distilled sterile water (blue bars) was added. Each bar represents the average of three independent experiments (*n* = 3), except for the medium control (*n* = 2). The error bars represent the standard deviation. The statistical differences between groups were evaluated using two-way ANOVA followed by the Šídák's multiple-comparison test (****, *P* < 0.0001; ***, *P* < 0.0001 to *P* < 0.001; **, *P* < 0.001 to *P* < 0.01; *, *P* < 0.01 to *P* < 0.05).

To investigate the impact of ethanol shock on the production of secondary metabolites and the relative concentration of other identified compounds in the culture, *S. venezuelae* NRRL B-65442 was cultivated in triplicates in MYM medium in the presence and absence of 6% ethanol added after 7 h of initial growth. The methanolic culture extracts were analyzed by LC-MS after 18 h, 24 h, and 48 h of incubation. The data set was compared using an untargeted data analysis workflow to identify the most significantly affected compounds. Ethanol shock was found to have a profound effect on the metabolite profile, and the amount of nearly all secondary metabolites detected was either increased or decreased (Fig. 1). The most obvious impact was on the hydroxamate siderophores because they are among the most abundant compounds already after 16 h of cultivation, and in the case of desferrioxamines/legonoxamines, they are represented by a large number of congeners and derivatives which all behaved identical qualitatively. The production of both foroxymithine and desferrioxamine B congeners were nearly abolished after the addition of ethanol to the medium. The same was true for the 2-hydroxyphenylthiazolines, such as thiazostatin A or B, which were however detected only at very low levels. Interestingly, the same very strong depletion was observed for PepX (Fig. 1), indicating a possible correlation in the regulation of the biosynthesis of this simple ribosomal peptide and the more complex multienzyme factories responsible for the synthesis of foroxymithine, desferrioxamine B, legonoxamine A, and the 2-hydroxyphenylthiazolines. In agreement with our previous observations, the production of chloramphenicol (13) and of the jadomycin-related compounds l-digitoxosyl-phenanthroviridin and phenanthroviridin aglycone was increased by ethanol shock but only at later time points. Conversely, the level of the gaburedins seemed to be reduced by ethanol shock at the later time points (Fig. 1).

In the search for other compounds which were affected and could help to unravel the regulatory network, several modified nucleosides stood out. Most of the tentatively identified nucleosides, namely, *N*,*N*-dimethyl-*O*-methyladenosine, two *N*,*O*-dimethyladenosine isomers, and threonylcarbamoyladenosine, were downregulated strongly upon ethanol shock, while an *N*,*N*-dimethyladenosine was upregulated strongly (Fig. 1). Although the fragmentation pattern allows only the localization of the methyl groups on either the base or sugar part without further details, it is likely that the methylation of adenine takes place at the *N*^6^ position. *N*^6^,*N*^6^,2′-*O*-trimethyladenosine, *N*^6^,2′-*O*-dimethyladenosine, *N*^6^-threonylcarbamoyladenosine, and *N*^6^,*N*^6^-dimethyladenosine are all well-known products of RNA modification reactions that play an important role in transcription and translation (29). However, while the “RNA epigenetic” system of enzymes responsible for “writing, erasing and reading” the RNA *N*^6^-methyladenosine (m^6^A) modifications is reasonably well established for many eukaryotes, little is known about such a system in bacteria despite m^6^A being confirmed as a widespread modification (29, 30). Also, the relatively high levels of these free, modified nucleosides are puzzling considering that only <0.08% of the adenosine in mRNA from the two Gram-positive bacteria, Staphylococcus aureus and Bacillus subtilis, was found to be *N^6^*-methylated (31).

Another compound produced in larger amounts after ethanol shock was *N*-phenylacetylglutamine (Fig. 1), an amino acid derivative known as a gut microbiota-host cometabolite that has been investigated as a potential biomarker for coronary heart disease (32). To the best of our knowledge, this compound has not yet been reported from streptomycetes and its biosynthetic origin is not clear. However, since the increase of *N*-phenylacetylglutamine coincides with the decrease in legonoxamine A content, it is possible that its production is not upregulated on a transcriptional or translational level but is influenced merely by the availability of larger amounts of phenylacetyl-coenzyme A (CoA) that is otherwise channeled into the biosynthesis of legonoxamine A.

We also observed that several amino acids and *N*-acetyl amino acids are more abundant upon ethanol shock. Since the amino acids are already present in the medium as nutrients in even larger amounts, their relatively higher concentration upon ethanol-induced stress might indicate lower uptake and consumption (Fig. 1). However, numerous other metabolites were not or only very little affected, as exemplified for the two cyclo(Phe-Pro) stereoisomers and biotin, demonstrating that the strong downregulation of several metabolites shown above cannot be simply explained by a general reduction in metabolism upon the ethanol shock.

In the course of a follow-up study, three additional batches of *S. venezuelae* NRRL B-65442 cultures in MYM medium in the presence and absence of 6% ethanol were analyzed. To test the robustness and reproducibility of the above-described findings, these data were examined by a targeted workflow, and the results are shown in Fig. S10 in the supplemental material. Despite strong variation in the peak areas between batches, all the trends reported above could be confirmed except for the gaburedins, for which no clear picture was obtained.

### Ethanol shock causes massive changes in the transcriptome.

**FIG 2:**
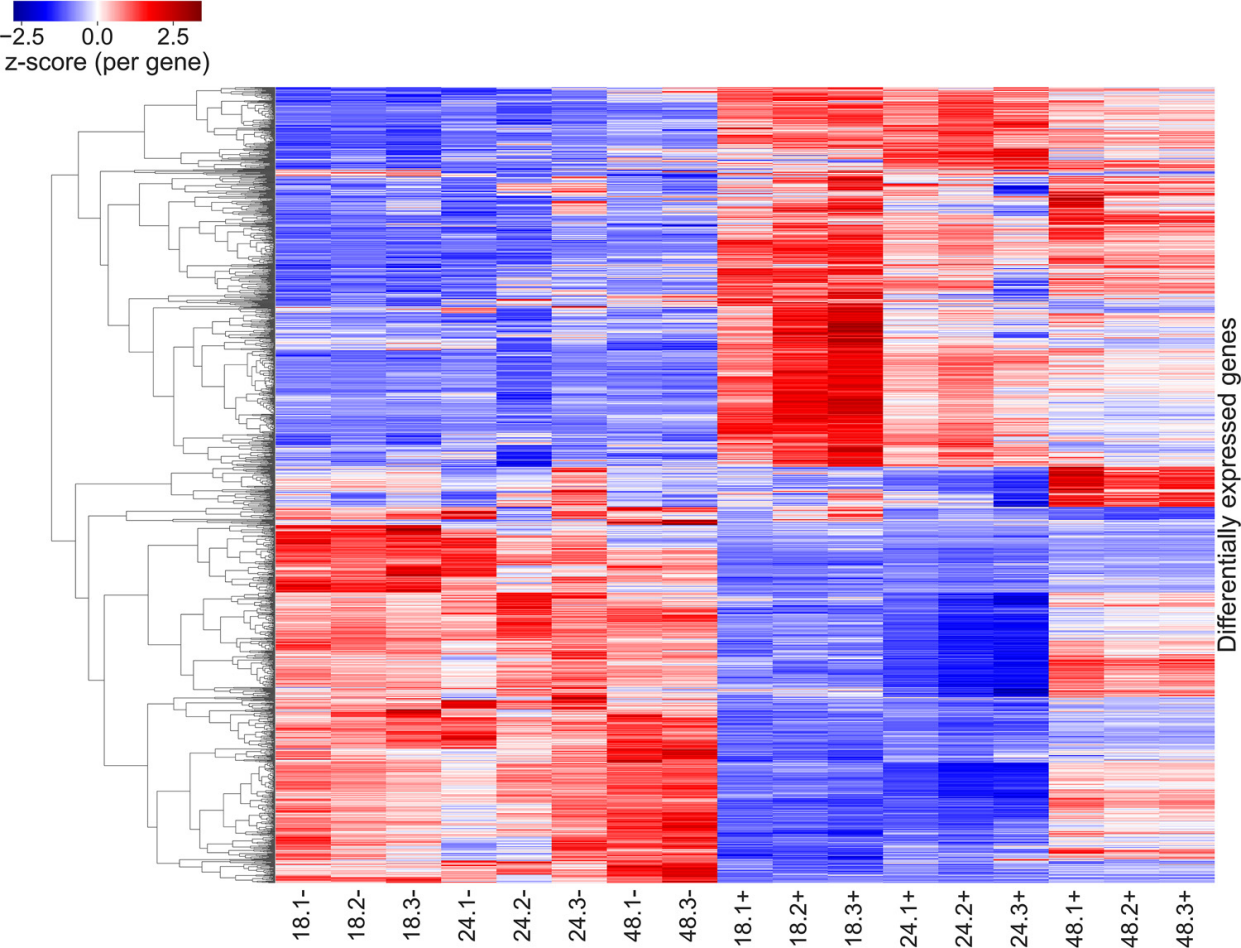
Comparison of differentially expressed genes across all samples and time points. Sample names on the bottom show the time point (18, 24, and 48 h) of collection, the ID of the biological replicate (1–3), and treatment with ethanol (+) or water (−). The individual cells each represent a differentially expressed gene in given sample. For each gene, a z-score is calculated based on mean and standard deviation across all samples. The Z-score is used to color the genes for a deviation from the mean with blue being less expressed, red being higher expressed, and white being expression close to the mean. Genes are clustered based on their correlation with each other, resulting in clear separation of ethanol-shocked and control samples.

A comparative RNA-seq-based transcriptome analysis was performed for the *S. venezuelae* cultures grown with and without ethanol shock. The samples for RNA isolation were taken from the same cultures for which secondary metabolome analysis have been performed. After an 18-h post-ethanol shock, 1,246 out of the 7,204 annotated genes in the *S. venezuelae* genome (17.2%) were found to be differentially affected. At 24 and 48 h postethanol shock, 1,115 and 638 genes, respectively, were differentially expressed (see Table S1 in the supplemental material). A comparison of differentially expressed genes across all samples and time points is shown as a heat map in Fig. 2, where it is evident that the most significant changes in the transcriptome happen at 18 h and 24 h post-ethanol shock. It was also evident that at the 48-h time point that the expression of many genes is more similar to that of the control samples, as already indicated by the reduced number of differentially expressed genes. A comparison of all differentially expressed genes over all samples and time points reveals that their expression pattern clearly separates ethanol shock samples from control samples. The three different time points show distinct gene sets up- or downregulated consistently within each of the three ethanol shock replicates, with more similarity between 18 h and 24 h.

The effect of ethanol treatment on gene expression has been studied for bacteria used to produce alcohol-containing beverages, such as Lactobacillus plantarum and Oenococcus oeni (33, 34). The most frequently observed response to environmental stress, including ethanol shock, was reported to be changes in the bacterial membrane. Ethanol and other chemicals, such as hydroxylamine derivatives, increase membrane fluidity (35), and this process can be counteracted via several mechanisms. They include changes in the phospholipid composition (36) as well as overexpression of membrane-binding heat shock proteins, such as DnaK and GroEL (37). We specifically looked at the transcription profiles of *S. venezuelae* genes RS17015 and RS22115 encoding DnaK and GroEL heat shock proteins, respectively. Both genes were upregulated at 18 h and 24 h, with RS22115 also upregulated at 48 h after ethanol shock. Besides being molecular chaperons that assist in correct protein folding, DnaK and GroEL are recruited to the bacterial membrane upon ethanol shock in Bacillus subtilis (37). At least for GroEL it was shown that such binding stabilizes the membrane (38). It is thus logical to assume that upregulation of DnaK and GroEL homologue expression in *S. venezuelae* upon ethanol shock serves the same purpose.

Some other proteins, such as Asp23 in Staphylococcus aureus overexpressed upon alkaline shock, were shown to be tethered to the membrane and implicated in stress response (39). Deletion of the *asp23* gene leads to upregulation of cell wall stress genes and increases resistance to the membrane-targeting antibiotic daptomycin (40). In *S. venezuelae*, expression of the gene RS07310 encoding an Asp23/Gls24 family envelope stress protein is downregulated at all three time points examined, which correlates well with the findings reported for its S. aureus counterpart.

From the data presented above, it appears plausible that the membrane of *S. venezuelae* undergoes significant alterations upon cell exposure to ethanol. One of the most important consequences of such perturbations could be changes in the state of membrane-bound proteins that may interact with ECFs and modulate their activity (41). With this in mind, we examined the effect of ethanol shock on the transcription of all 56 σ factors encoded in the *S. venezuelae* genome at 18 h, 24 h, and 48 h. The results of this analysis are presented in Table 2. In total, the expression of 14 σ factors, with 10 of them ECFs, was affected by the ethanol shock, with 2 being downregulated (1 of them ECF) and 4 upregulated (3 of them ECFs) at all three time points. We suggest that σ factors, whose expression was affected only at 24 h or/and 48 h and not at 18 h, are unlikely to be involved in the regulation of secondary metabolism since metabolome changes after ethanol shock were observed already at 18 h.

**TABLE 2:** Expression of *S. venezuelae* σ factors affected by the ethanol shock^*a*^

Gene	Expression at:			σ domains
	18 h	24 h	48 h	
RS00060			Up	r2, r4
RS02270	Down	Down		r2, r3, r4
RS13895	Up	Up	Up	r2, r3, r4
RS15140	Up	Up		r1, r2, r3, r4
RS15760	Down	Down	Down	r2, r4
RS15950	Up	Up	Up	r2, r4
RS17255		Up		r2, r4, SnoaL
RS18620	Up			r2, r4
RS18755	Down	Down	Down	r2, r3, r4
RS21030	Up			r2, r4
RS22475		Down		r2, r4
RS22785	Up	Up	Up	r2, r4
RS23885	Up			r2, r4
RS24280	Up	Up	Up	r2, r4

a Cells representing genes that encode ECFs are shaded in gray.

### Correlation of the metabolome and transcriptome data does not always provide consistent results.

We attempted to correlate the metabolome and transcriptome data, specifically looking at the genes known to be involved in the biosynthesis of detected secondary metabolites. Genes for the biosynthesis of desferrioxamine, legonoxamines, and foroxymithines, as well as the gene encoding peptide X, were clearly downregulated at the 18-h and/or 24-h time points, which correlated well with the drastically reduced accumulation of these compounds, while no such correlation was found for gaburedins. The finding that there was no correlation for gaburedins may be explained by the upregulation of the biosynthesis at the level of translation or via a decreased availability of the precursor γ-aminobutyric acid (19). The correlation between the transcriptome and metabolome was also poor for chloramphenicol and jadomycin BGCs, as none of the genes involved in the biosynthesis of corresponding scaffolds (e.g., nonribosomal peptide synthetase [NRPS] for chloramphenicol and ketosynthase for jadomycin) were affected by the ethanol shock. However, the expression of the regulatory genes *jadR1* (RS29820) and *jadR* (RS29940), as well as some of the biosynthetic genes, namely, *jadJ* (RS29825, acyl-CoA carboxylase), *jadB* (RS29840, ketosynthase chain-length factor), *jadF1* (RS29860, FAD-dependent oxidoreductase), *jadH* (RS29870, FAD-dependent oxidoreductase), and *jadK* (RS29875, alpha/beta hydrolase) in the jadomycin BGC, were all upregulated at 18 h. It is possible that enzymes encoded by the upregulated *jad* genes represent “bottlenecks” in the jadomycin biosynthetic pathway, and hence, their overexpression circumvents this problem.

In the case of chloramphenicol, the situation appears to be more complex. The *cmlB* gene (RS04460) encoding aminodeoxychorismate (ADC) synthase is downregulated at the 24-h time point after the ethanol shock, which does not correlate with the apparent increase in chloramphenicol production. ADC synthase, besides chloramphenicol biosynthesis, is involved in the formation of *para*-aminobenzoic acid, a precursor for the formation of folates, the essential cofactors. Chang et al. (42) reported cloning of the *S. venezuelae* ISP5230 (ATCC 10712) PABA biosynthesis genes, *pabA* and *pabB*, which are located outside the chloramphenicol BGC and are responsible for the ADC synthesis. Disruption of *pabA* resulted in 97% reduction in the chloramphenicol biosynthesis, clearly suggesting that CmlB is not the main enzyme responsible for the generation of ADC starter. In *S. venezuelae* NRRL B-65442, homologous proteins are encoded by the genes RS31140 (*pabA*) and RS31145 (*pabB*), and their expression is not affected by the ethanol shock. Hence, downregulation of *cmlB* is unlikely to have any consequences for the chloramphenicol production. On the other hand, expression of the gene *cmlJ* (RS04490) encoding an SDR family oxidoreductase implicated into the last step of chloramphenicol biosynthesis (21) is upregulated at 24 h, which correlates well with the increase of this antibiotic yield after 24 h (Fig. 1). Apart from the regulation of protein expression or enzyme function, increased levels of chloramphenicol’s main precursor, chorismate, could explain the observed stimulation of the biosynthesis. Our data show an increased expression of chorismate biosynthesis genes (see Fig. 5B), providing support for this hypothesis.

Notably, chorismate is the precursor for the biosynthesis of both chloramphenicol and thiazostatins/watasemycins (20). Hence, the suppression of the thiazostatins biosynthesis upon ethanol shock is likely due to the depletion of chorismate that may be channeled into the chloramphenicol biosynthesis pathway since no changes in the expression of the watasemycin biosynthetic genes could be detected.

Although the metabolome analysis did not detect other compounds which, according to the earlier reports could have been produced by *S. venezuelae*, we examined the gene expression patterns of their BGCs upon ethanol shock. According to such an analysis, the expression genes for ectoin, geosmin, and 2-methylisoborneol biosynthesis, as well as the PKS genes from the spore pigment BGC, were downregulated upon ethanol shock (Table S1). These compounds are likely produced in the culture but not detected by the applied metabolomics workflow due to their high polarity (ectoin), volatility (geosmin and 2-methylisoborneol), or low solubility (spore pigment).

### Genome and omics-based analyses of selected *S. venezuelae* metabolic pathways.

**FIG 3:**
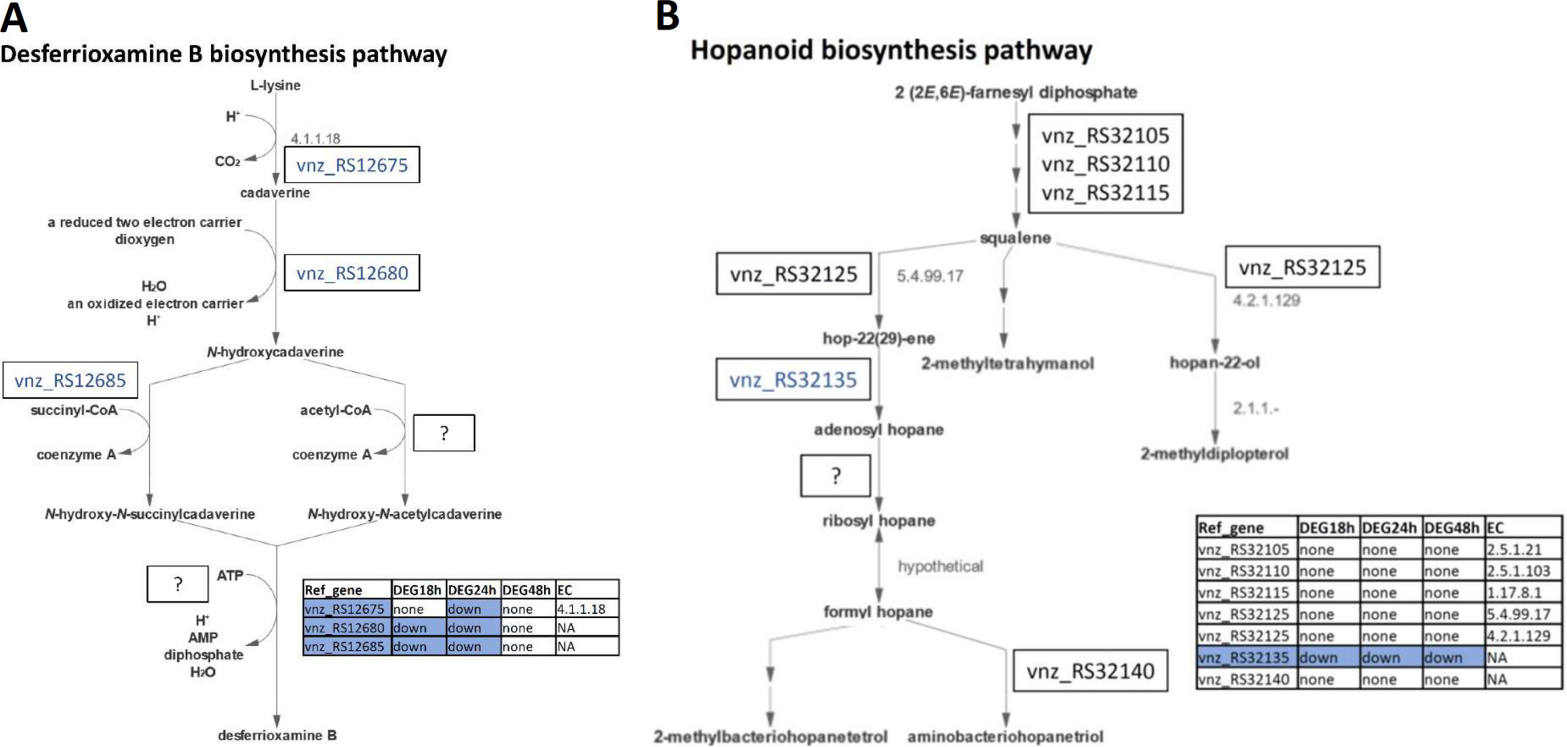
(A) Visualization of the desferrioxamine B biosynthesis pathway according to MetaCyc (PWY-6376). (B) Visualization of the hopanoid biosynthesis pathway according to MetaCyc (PWY-7072). *S. venezuelae* genes encoding enzymes involved in the pathways and their expression patterns at 3 time points after ethanol shock are shown. Downregulated genes are shown in blue.

**FIG 4:**
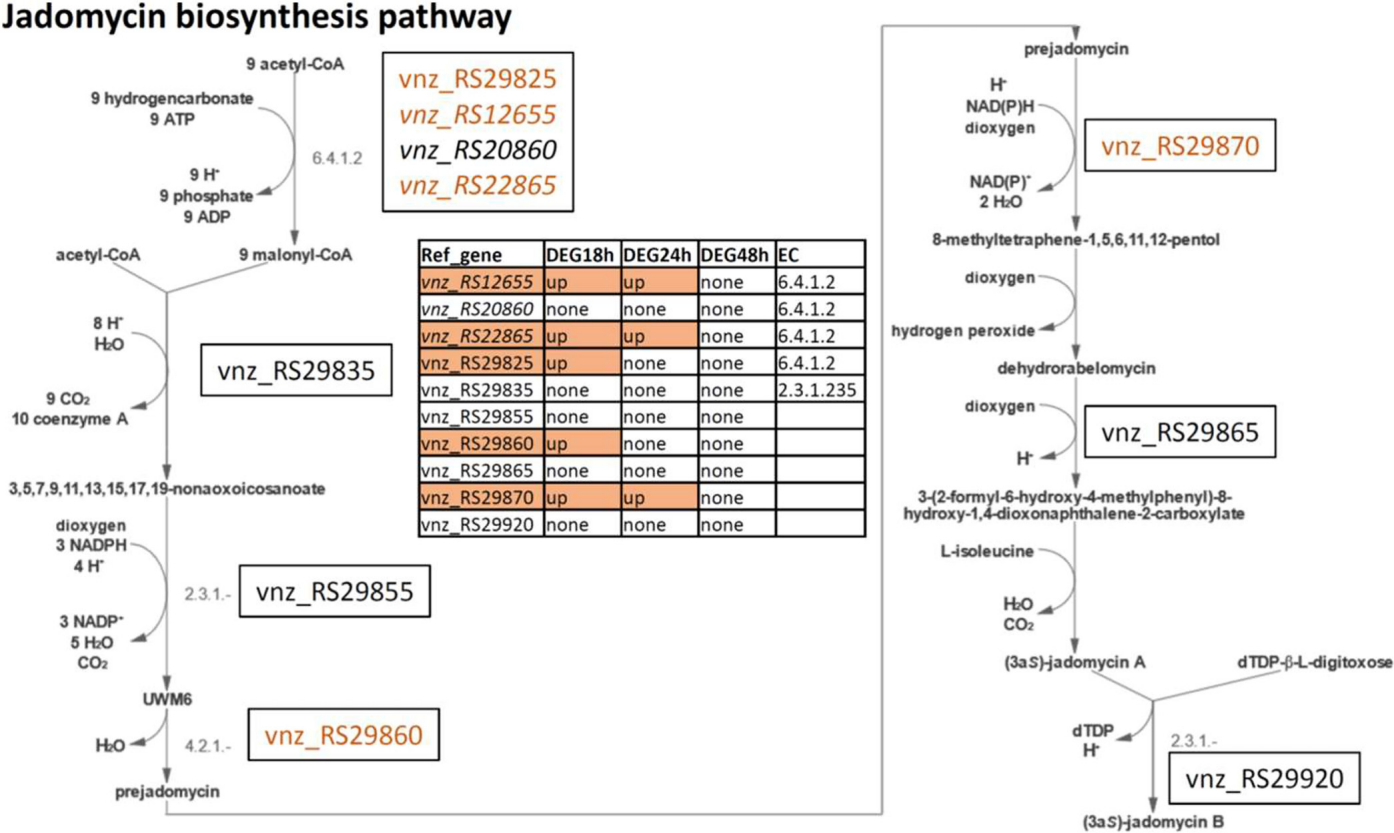
Visualization of the jadomycin biosynthesis pathway according to MetaCyc (PWY-6679). Genes encoding enzymes involved in the pathways and their expression patterns at 3 time points after ethanol shock are shown. Upregulated genes are shown in orange.

**FIG 5:**
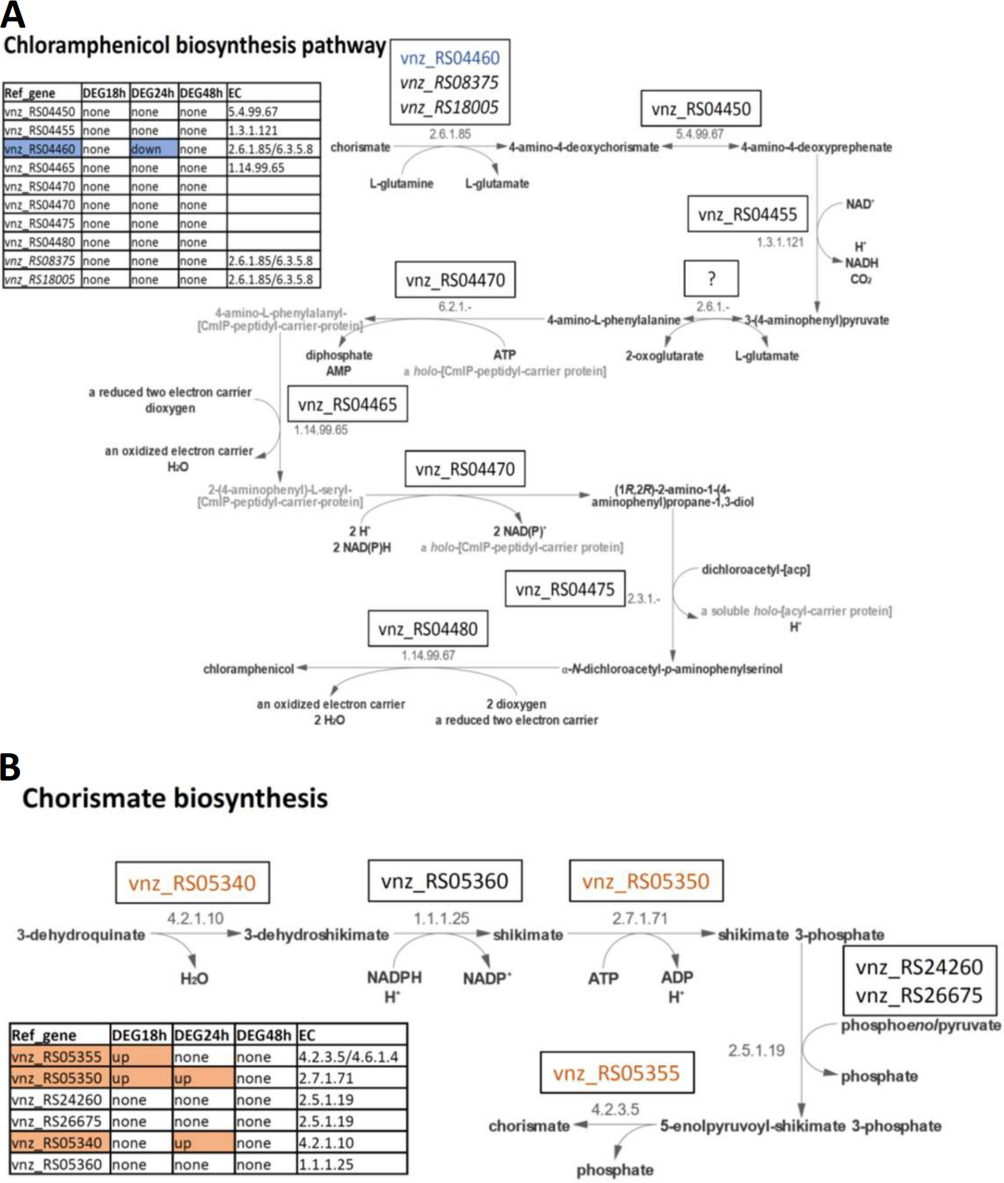
(A) Visualization of the chloramphenicol biosynthesis pathway according to MetaCyc (PWY-8032). (B) Visualization of the chorismate biosynthesis pathway according to MetaCyc (PWY-6163). Genes encoding enzymes involved in the pathways and their expression patterns at 3 time points after ethanol shock are shown. Downregulated genes are shown in blue and upregulated in orange.

To better correlate the transcriptome and metabolome data for the secondary metabolite biosynthesis pathways affected by the ethanol shock in *S. venezuelae*, we performed their integration into the schemes presented in Fig. 3 to 5A and in Fig. S11 in the supplemental material. Figure 3A shows the desferrioxamine B biosynthesis pathway in MetaCyc (PWY-6376), overlaid with differential gene expression data for all four genes from the desferrioxamine BGC. The gene which product catalyzes the first reaction, conversion of l-lysine to cadaverine is downregulated at the 24-h time point, while the genes governing the next two reactions, up to *N*-hydroxy-*N*-succinylcadaverine, are also downregulated at 18 h. Only the last gene encoding the DesD enzyme responsible for the condensation of *N*-acylated *N*-hydroxycadaverine with two molecules of *N*-succinyl-*N*-hydroxycadaverine to form various desferrioxamines, including desferrioxamine B, was not differentially expressed.

Figure 3B shows the hopanoid biosynthesis pathway in MetaCyc (PWY-7072), overlaid with the differential gene expression data. The pathway from (2E,6E)-farnesyl diphosphate to squalene requires the products of three genes, but the exact reactions involved are unclear based on the predictions. The pathway assumes the conversion of presqualene diphosphate to squalene as two separate reactions via hydroxysqualene. Only for the second step, a gene coding for the enzyme responsible was predicted. However, a different gene was predicted to specify a one-step reaction from presqualene diphosphate to squalene, which is attributed to the epoxysqualene pathway. Furthermore, only some of the branches of the pathway are predicted to have genes encoding enzymes catalyzing these reactions, ending at hopan-22-ol and aminobacteriohopanetriol. The second branch contains the only reaction that is connected to a differentially expressed gene, RS32135, which is downregulated at all time points after the ethanol shock. This gene encodes adenosylhopane synthase HpnH that is responsible for the synthesis of C35 bacteriohopanepolyols, which represent an important part of bacterial membranes largely affecting their fluidity (43). Hence, downregulation of this gene likely results in a more rigid and less permeable membrane, thus adding to the stabilizing effect of DnaK/GroEL upregulation and Asp23 homologue downregulation.

The jadomycin biosynthesis pathway in MetaCyc (PWY-6679) is shown in Fig. 4, overlaid with differential gene expression data. All reactions in this pathway were matched with particular genes in the jadomycin BGC. Genes for enzymes catalyzing three different reactions were upregulated in this pathway; RS29860 is upregulated at 18 h and corresponds to the reaction from UWM6, an angucycline intermediate, to prejadomycin. The follow-up reaction is governed by RS29870, which is upregulated also at 24 h. Finally, the first reaction in the pathway, namely, the conversion of acetyl-CoA to malonyl-CoA, is predicted to be enabled by four different genes, of which only one (RS29825) belongs to the jadomycin BGC and is upregulated at 18 h. The other three genes are not located within jadomycin BGCs, but two of them are upregulated at 18 h and 24 h, with the last one not being differentially expressed.

Figure 5A shows the chloramphenicol biosynthesis pathway according to MetaCyc (PWY-8032), overlaid with differential gene expression data. This pathway consists of nine reactions catalyzed by the enzymes encoded by seven genes of the chloramphenicol BGC. One reaction [3-(4-aminophenyl)pyruvate to 4-amino-l-phenylalanine] does not have a gene prediction, as the MetaCyc database does not have a representative enzyme or sequence attributed to it. Out of the seven genes, only one is differentially expressed; RS04460 is downregulated at 24 h and corresponds to the first step of the pathway.

### Several central metabolic pathways are affected by ethanol shock.

**FIG 6:**
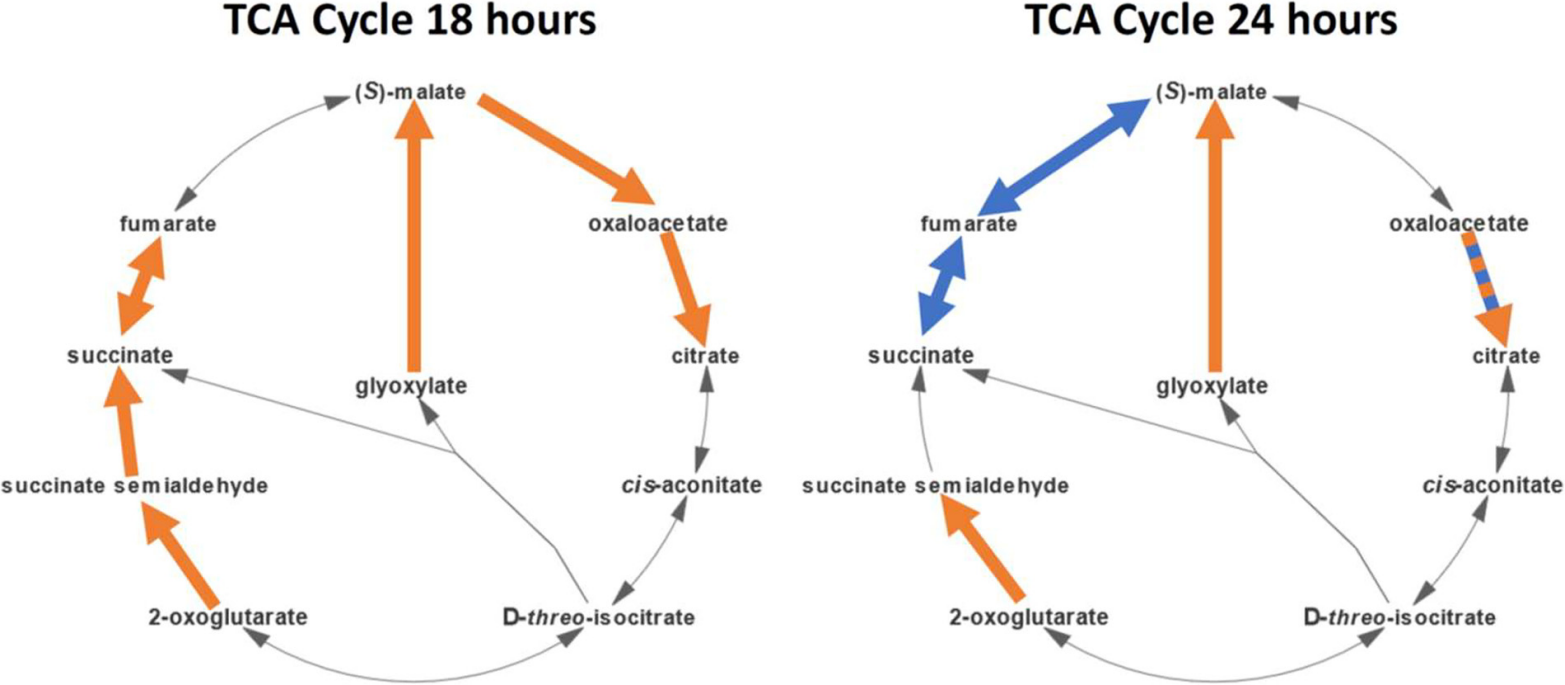
Reactions in the TCA cycle and glyoxylate shunt colored according to the gene expression state at 18 h and 24 h after ethanol shock. Reactions where at least one gene encoding an involved enzyme is upregulated are colored orange, and downregulated genes are colored blue. The reaction converting oxaloacetate to citrate can be catalyzed by the enzyme products of one up- and one downregulated gene and is colored both orange and blue.

Besides the genes governing the biosynthesis of secondary metabolites, ethanol shock caused significant changes in the expression of genes involved in central metabolic pathways of *S. venezuelae* that are responsible for the generation of energy and primary metabolites. The tricarboxylic acid (TCA) cycle in *S. venezuelae*, schematically shown in Fig. 6 and, in more detail, in Fig. S11, includes a glyoxylate shunt. The glyoxylate shunt has been shown to be connected to oxidative and antibiotic stress response in various bacteria, but no clear understanding of its significance has yet been obtained (44). The gene expression profile shows an upregulation of the genes whose products are responsible for most parts of the TCA cycle at 18 h after the ethanol shock (genes RS12505, RS22220, RS22405, RS22565, RS22570, RS24745, RS26530, and RS30430). At 24 h, the expression profile changes drastically, as the genes for the reactions from succinate to malate are downregulated (genes RS01060 and RS34050) and most genes that were previously upregulated are now not differentially expressed, leaving only RS12505, RS24745, and RS30430 expressed at a higher level. In addition, RS12505, whose product encodes citrate synthase responsible for the conversion of oxaloacetate to citrate, is upregulated at 18 and 24 h, while two other genes annotated as coding for “citrate synthase 2” (RS20910) and “bifunctional 2-methylcitrate synthase/citrate synthase” (RS27870) are not differentially expressed or downregulated, respectively. Most likely, the products of the genes RS20910 and RS27870 are not involved in the TCA. In the glyoxylate shunt, one of the genes involved in this pathway, namely, RS30430, is upregulated at 18 h and 24 h (Fig. 6, Table S1). At 48 h after ethanol shock, only a single gene involved in the TCA cycle is differentially expressed, RS24745, whose product is responsible for the conversion of 2-oxoglutarate to succinate semialdehyde. An upregulated TCA cycle, together with the glyoxalate shunt could thus provide increased amounts of NADH, NADPH, and coenzyme A, which are all known as cofactors of enzymes involved in the biosynthesis of secondary metabolites.

**FIG 7:**
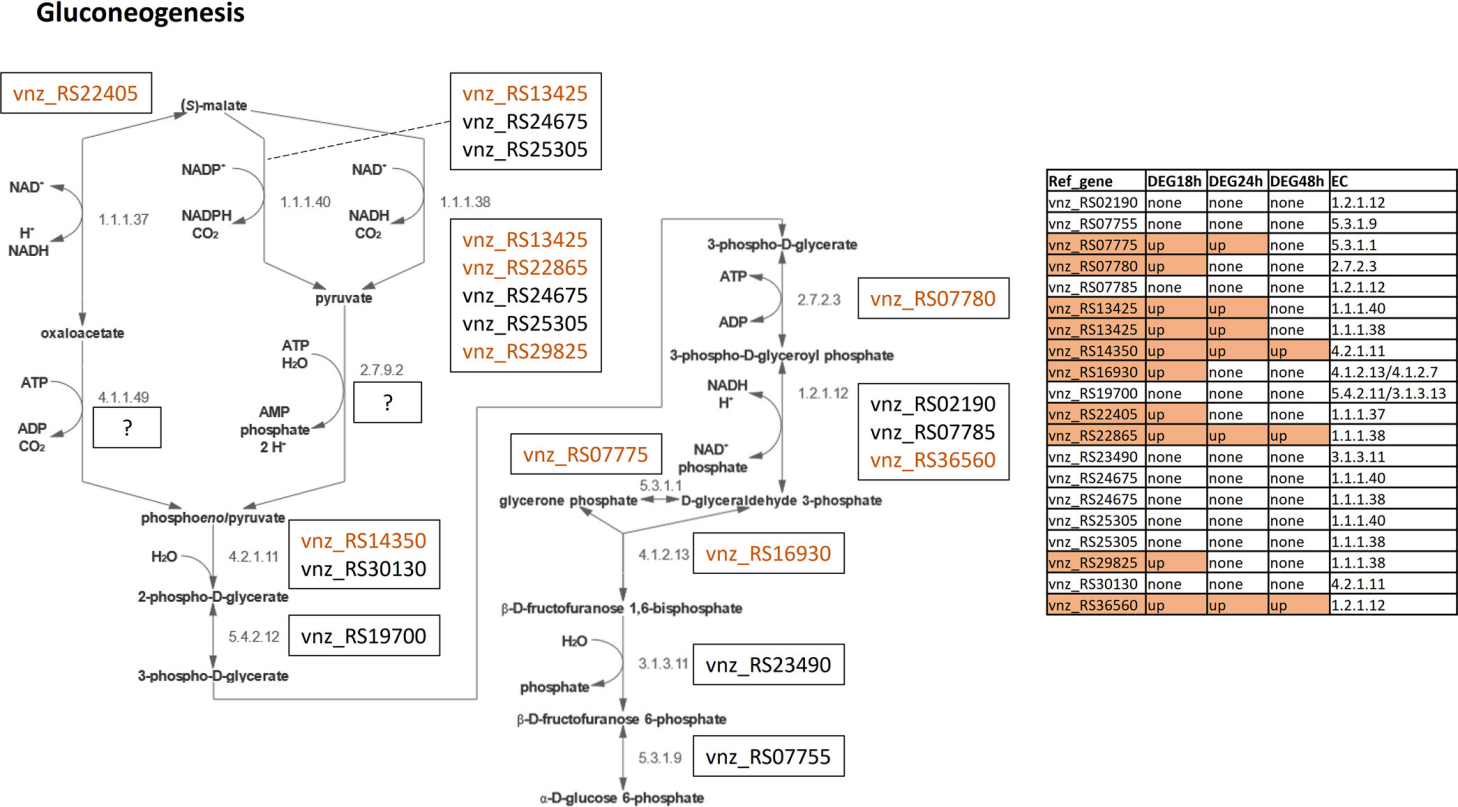
Visualization of the gluconeogenesis pathway according to MetaCyc (GLYCOLYSIS). *S. venezuelae* genes encoding enzymes involved in the pathways and their expression patterns at 3 time points after ethanol shock are shown. Genes are colored by their status of differential gene expression, namely, orange for higher expression and blue for lower expression.

Several genes whose products catalyze reactions in gluconeogenesis (Fig. 7) were also found to be upregulated upon ethanol shock, with 9 genes (RS07775, RS07780, RS13425, RS14350, RS16930, RS22405, RS22865, RS29825, and RS36560) at 18 h after ethanol shock, dropping to 5 (RS07775, RS13425, RS14350, RS22865, and RS36560) at 24 h, and only 3 (RS14350, RS22865, and RS36560) at 48 h (Table S1). Gluconeogenesis is linked to the pentose phosphate pathway, which generates NADPH, an essential cofactor needed for anabolic processes, including the biosynthesis of secondary metabolites. Hence, upregulation of gluconeogenesis may boost secondary metabolism by supplying NADPH that can be utilized in pathways encoded by upregulated BGCs, such as the one for jadomycin biosynthesis (Fig. 4).

**FIG 8:**
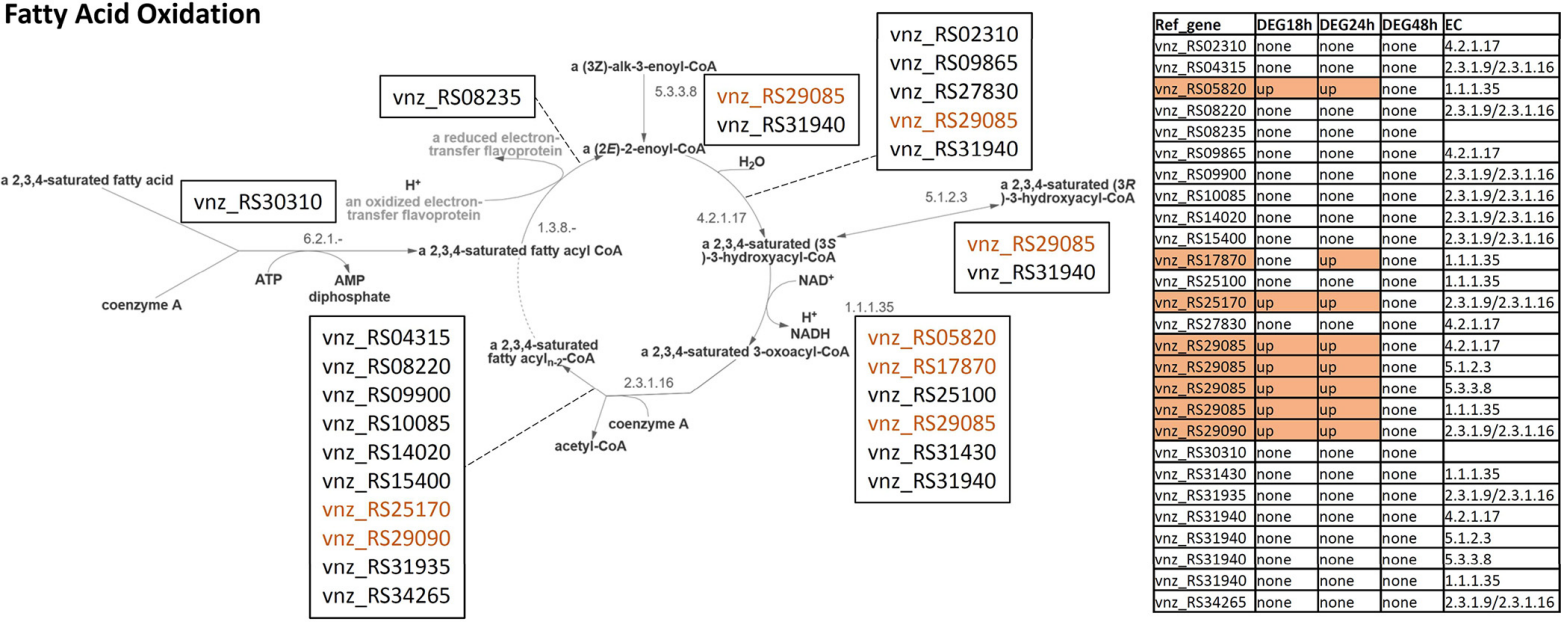
Visualization of the fatty acid oxidation pathway according to MetaCyc (FAO-PWY). *S. venezuelae* genes encoding enzymes involved in the pathways and their expression patterns at 3 time points after ethanol shock are shown. Genes are colored by their status of differential gene expression, namely, orange for higher expression and blue for lower expression.

Acetyl-CoA is one of the central primary metabolites and is also used as a precursor or building block in the polyketide biosynthesis. The main catabolic pathway leading to acetyl-CoA is fatty acid oxidation, which according to MetaCyc involves up to 27 genes in *S. venezuelae* (Fig. 8). Four (RS05820, RS25170, RS29085, and RS29090)/five (RS17870) of these genes are upregulated at 18/24 h after the ethanol shock. The increased pool of acetyl-CoA may supply precursors for jadomycin biosynthesis, further boosting this process along with the overexpression of biosynthetic genes.

We also took a closer look at the expression profiles of the genes involved in the biosynthesis of chorismate, which is a precursor for both tryptophan and chloramphenicol, with chloramphenicol being overproduced upon ethanol shock (Fig. 1). Chorismate biosynthesis is accomplished via five reactions, starting from 3-dehydroquinate (Fig. 5B). Three of the five reactions are catalyzed by the enzymes encoded by genes whose expression is upregulated after ethanol shock. The upregulated genes are RS05355 at 18 h, assigned to the conversion of 5-enolpyruvoyl-shikimate 3-phosphate to chorismate; RS05340 at 24 h, assigned to the reaction from 3-dehydroquinate to 3-dehydroshikimate; and RS05350 at 18 and 24 h, assigned to the reaction from shikimate to shikimate 3-phosphate. Considering this information, it is plausible to assume that the boost in chloramphenicol biosynthesis, in the absence of upregulation of its BGC, can be caused by the increased pool of chorismate. Accumulation of tryptophan upon ethanol shock can be caused by a reduced uptake of this amino acid from the medium, counteracted by an increased chorismate pool and overexpression of tryptophan biosynthesis genes to satisfy cellular demand for this amino acid. In the tryptophan pathway, 2 genes were found to be upregulated, namely, RS08410 (encoding phosphoribosyl anthranilate isomerase) at 18 h and RS08355 (encoding indole-3-glycerol phosphate synthase), at 24 h.

**FIG 9:**
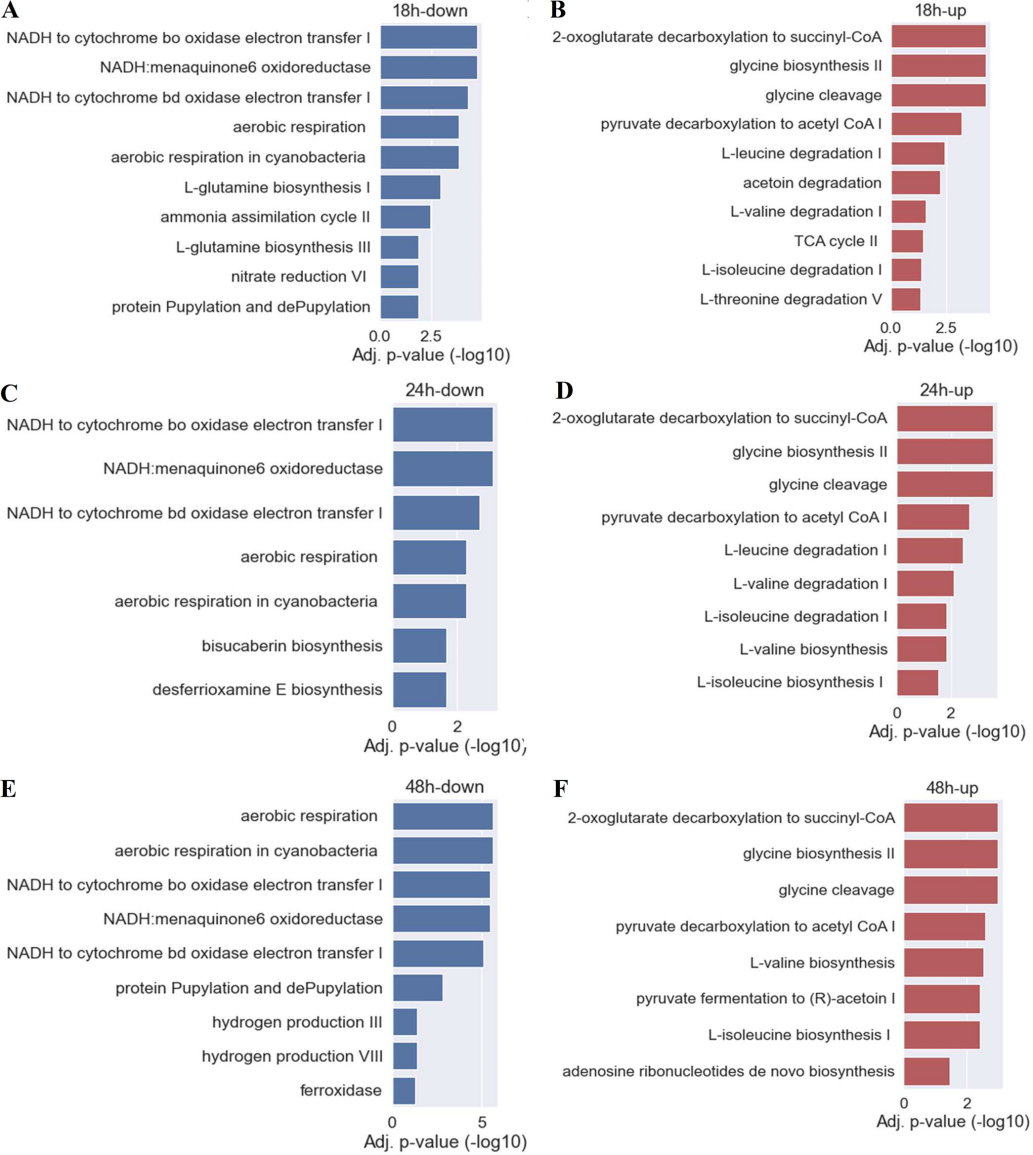
Enriched pathways in differentially expressed genes. Show pathways enriched in downregulated genes (A, C, E) and in upregulated genes (B, D, F), for 18 h, 24 h, and 48 h after ethanol shock. Only pathways with an adjusted *P* value (Benjamini-Hochberg correction) below 0.05 are shown.

To have an indication of what the changes in the transcriptome might mean for the general cellular metabolism, we carried out a gene set enrichment analysis on several predicted pathways affected by ethanol shock (Fig. 9). The differentially expressed genes for each time point were used to generate a list of pathways up- or downregulated after the ethanol shock. The number of enriched pathways ranges from 10 to 7. Across all time points, 2-oxo acid dehydrogenase complexes and amino acid metabolism pathways were upregulated (Fig. 9B, D, F). The only class of pathway enriched in downregulated genes across all samples was aerobic respiration (Fig. 9A, C, E). At 18 h, also the glutamine biosynthesis, ammonia assimilation, and nitrate reduction pathways were downregulated. The related pathways for hydroxamate siderophores biosynthesis (bisucaberin and desferrioxamine) were downregulated at 24 h. Lastly, the protein Pupylation and dePupylation pathways were enriched at 18 h and 48 h, respectively. Notably, all genes involved in protein Pupylation and dePupylation (Pup: Prokaryotic Ubiquitin-like Protein) were statistically significantly downregulated at all three time points. That this pathway, containing only three genes, does not show up at 24 h is due to the higher number of genes being downregulated at this time point, leading to it not reaching the *P* value threshold for the enrichment. Protein Pupylation has been shown to be vital to oxidative stress response in Streptomyces coelicolor, while the proteasome was not (45, 46), and a *pup* deletion mutant was demonstrated to produce substantially smaller amounts of two pigmented antibiotics (46).

In conclusion, in this study, we demonstrated a complex response of *S. venezuelae* NRRL B-65442 to ethanol shock, including massive changes in the transcriptome which are reflected in the metabolome. We hypothesize that these changes are due to the alterations in the membrane via stress response mechanisms aimed at counteracting the membrane fluidization caused by ethanol. New insights in the stress response in *Streptomyces* bacteria, especially in connection with the secondary metabolite biosynthesis, will help to design new strategies for the metabolic engineering of these industrially important bacteria aimed at increasing the production of medically and agriculturally important natural products.

## MATERIALS AND METHODS

### Growth conditions, fermentation, and ethanol shock.

Streptomyces venezuelae NRRL B-65442 was grown on ISP4 agar medium (Difco) at 28°C. Seeding cultures for fermentations were prepared by inoculation of 10 μL of a spore suspension in 10 mL of liquid TSB medium (Ovoid) and incubation in 150-mL Erlenmeyer flasks for 16 h at 28°C with shaking (220 rpm/min). Next, 2.5 mL (10% vol/vol) of seed culture was used to start a 25-mL fermentation in MYM medium, containing 2.1 g/L MOPS (Sigma). Ethanol shock was performed after 7 h of cultivation by adding 1.5 mL (6% vol/vol) of absolute ethanol or 1.5 mL distilled sterile water (control) to the cultures. After ethanol shock, the fermentations continued for 18, 24, and 48 h. All the fermentations were performed in triplets.

### Preparation of samples for RNA-seq.

The fermentations were stopped after 18, 24, and 48 h. Next, 1.5-mL samples were immediately taken from each culture and transferred in sterile RNase-free tubes containing 3 mL of RNAprotect bacterial reagent (Qiagen, Germany). The samples were vortexed for 10 sec, rested on a table for 5 min, transferred into 2.5-mL sterile RNase-free Eppendorf tubes and centrifuged for 12 min at 4,000 rpm and 4°C. Supernatants were removed and cells were frozen immediately at 80°C. One tube with the cells per sample was taken for RNA-seq analysis.

### Preparation of extracts for LC-MS.

After sampling for RNA-seq, the remaining cultures were freeze-dried and stored at −20°C until extraction. A total of 25 mL of methanol as added to the freeze-dried cultures and incubated for 60 min at a rotary shaker at 200 rpm. The mixtures were centrifuged for 10 min at 12,000 rpm, and the supernatants were transferred to new flasks. The methanol was removed using Rotavapor, and the dry residue was resuspended in 1 mL of methanol. A total of 100 μL of the extracts was used for the LC-MS analyses.

### LC-MS analyses and data interpretation.

LC-MS analyses of the obtained extracts were performed on a Vanquish Horizon ultra-high-performance liquid chromatography (UHPLC) system (Thermo Fisher Scientific) coupled to the electrospray ionization (ESI) source of an LTQ Orbitrap Velos mass spectrometer (Thermo Fisher Scientific). Separation was carried out on an Acclaim 120 C_18_, 2.1- by 150-mm, 3-μm high-performance liquid chromatography (HPLC) column (Thermo Fisher Scientific) using water and acetonitrile, both modified with 0.1% formic acid, as mobile phase A and B, respectively. The sample components were separated and eluted with a linear gradient from 5% to 95% B for 45 min followed by an isocratic column cleaning (9.5 min at 95% B) and re-equilibration step (10 min at 5% B). The flow rate was 0.45 mL/min, and the column oven temperature was set to 25°C.

High-resolution ESI-MS spectra were recorded in positive ion mode with a Fourier-transform (FT) resolution of 60,000. High- or low-resolution ESI-MS/MS spectra of the three most intense precursor ions in each MS^1^ spectrum were obtained in automated data-dependent acquisition mode using helium as the collision gas and the following settings: activation type, CID; isolation width, Δ*m/z *= 3; normalized collision energy of 35.0; activation Q, 0.250; and activation time, 30 ms. For selected samples, negative ion mode data were recorded in addition to assist with the dereplication.

The sum formulas of the detected ions were determined using Thermo Xcalibur 4.1.31.9 Qual Browser based on the mass accuracy (Δ*m/z*, ≤5 ppm) and isotopic pattern. Dereplication was accomplished with the aid of GNPS Library Search (47), the Dictionary of Natural Products 30.2 (CRC Press, Taylor & Francis Group), and CAS SciFinder (American Chemical Society). MZmine 2 was used for untargeted and Skyline 21.1.0.146 for the more accurate targeted comparison of the peak intensities between different samples or groups of samples (48, 49). The statistical differences between groups were evaluated using the two-way analysis of variance (ANOVA) followed by the Šídák's multiple-comparison test (using Prism 9.4.1; GraphPad Software).

### Differential gene expression analysis.

Prinseq-lite (50) v0.20.4 is used to filter the raw reads (parameters: “trim_qual_right 28, min_len 75, min_qual_mean 25”). A public reference genome was retrieved from RefSeq under accession NZ_CP018074.1 (Streptomyces venezuelae strain NRRL B-65442 genome), including the annotation information downloaded in a separate gff3 file. BWA-MEM (51) v0.7.16a with default parameters was used to align the reads to the reference. Aligned reads were separated from unaligned reads using SAMtools (52) sort (v1.15.1). From HTSeq (53) v2.0.1, the “htseq-count” function was used with the parameter “—nonunique=none” to count the aligned reads at each of the features in the gff3 reference file. Unaligned reads were analyzed with Kraken2 (54) v2.1.2, using the standard 8GB Kraken 2 Database built in 2019 from the NCBI REFSEQ bacteria, archaea, and viral libraries and the GRCh38 human genome (https://benlangmead.github.io/aws-indexes/k2) to explain their origin and check for contamination.

EdgeR (55) v3.36.0 and DESeq2 (56) v1.34.0 were used to predict differentially expressed genes. In EdgeR, the “TMM” method was used to normalize the expression values, and a quasi-likelihood F-test was performed to calculate *P* values. For DESeq2, the DESeq function was used with default parameters. All genes were analyzed by each of the two tools individually and considered differentially expressed with a fold change less than −0.6 or greater than 0.6 and a *P* value less than 0.05. The results were combined by considering only consensus predictions as differentially expressed, and conflicting results were deemed not differentially expressed.

### Pathway analysis of biosynthetic gene clusters.

Gapseq (57) was used to predict pathways and build a draft metabolic network from the reference genome. The genes from the RefSeq annotation were matched with genes predicted by gapseq via location. Genes without an exact location match were matched via overlap; further inspection ensured unique mapping and an overlap of >95% in both gapseq- and RefSeq-predicted genes.

Gene, reaction, and pathway associations were extracted from the generated draft metabolic network. Some reactions predicted by gapseq were not included in its metabolic network formulation database. Gene, reaction, and pathway associations for those genes were extracted from the reaction and pathway prediction output files of gapseq. Selection criteria were had a “good_blast” label and a blast bitscore of >200, similar to the parameters used in gapseq metabolic network creation.

The association of pathway identities (IDs) with biosynthetic gene clusters was performed by manual inspection of predicted reactions and pathways of genes located in biosynthetic gene clusters. Further reactions encoded by genes outside these clusters were found by searching for associations to the selected pathway IDs. Visualization of pathways was done using the pathway information and representation of the MetaCyc database and website (58).

### Data availability.

All RNA-seq reads generated and used in this study were deposited in the NCBI Sequence Read Archive under BioProject PRJNA888945.
