# Supplementary material for: Targeted Metabolomics and High-Throughput RNA Sequencing-Based Transcriptomics Reveal Massive Changes in the Streptomyces venezuelae NRRL B-65442 Metabolism Caused by Ethanol Shock
Source: Microbiol Spectr. 2022 Oct 31;10(6):e03672-22. doi: 10.1128/spectrum.03672-22 (PMC9769785; doi:10.1128/spectrum.03672-22)
Supplement: Supplemental file 1 — Supplemental material. Download spectrum.03672-22-s0001.pdf, PDF file, 2.8 MB [file spectrum.03672-22-s0001.pdf]

Supplemental Material

Targeted Metabolomics and RNAseq-based Transcriptomics Reveal Massive Changes in the *Streptomyces venezuelae* NRRL B-65442 Metabolism Caused by Ethanol Shock

Olga N. Sekurova[a#], Martin Zehl[b#], Michael Predl[cd], Peter Hunyadi[c], Thomas Rattei[cd], Sergey B. Zotchev[a]

[a]Department of Pharmaceutical Sciences, Division of Pharmacognosy, University of Vienna, 1090 Vienna, Austria

[b]Department of Analytical Chemistry, Faculty of Chemistry, University of Vienna, 1090 Vienna, Austria

[c]University of Vienna, Centre for Microbiology and Environmental Systems Science, Division of Computational System Biology, 1030 Vienna, Austria

[d]University of Vienna, Doctoral School in Microbiology and Environmental Science, 1030 Vienna, Austria

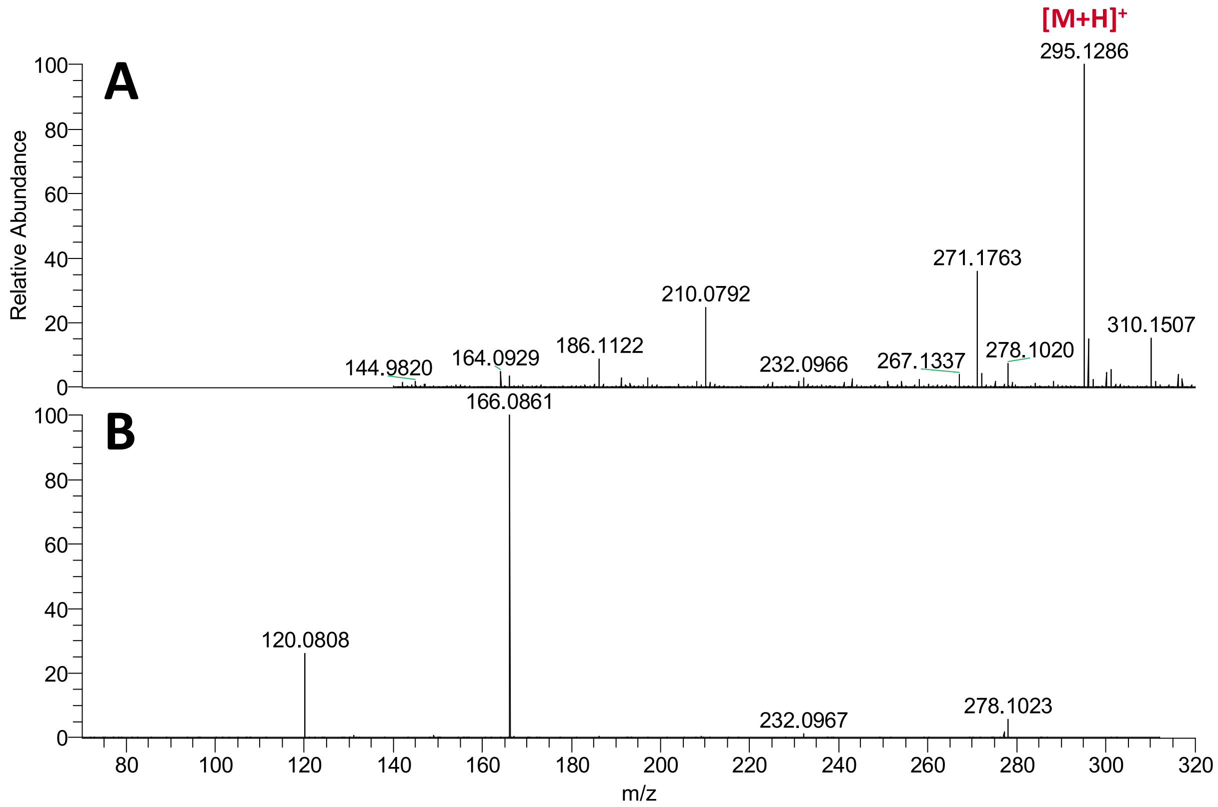

Figure S1A. High resolution ESI mass spectrum of gaburedin A (A) and high resolution MS/MS spectrum of its $[M+H]^+$ ion at *m/z* 295.1286 (B). Reference MS/MS data can be found in https://doi.org/10.1039/C3SC52536H.

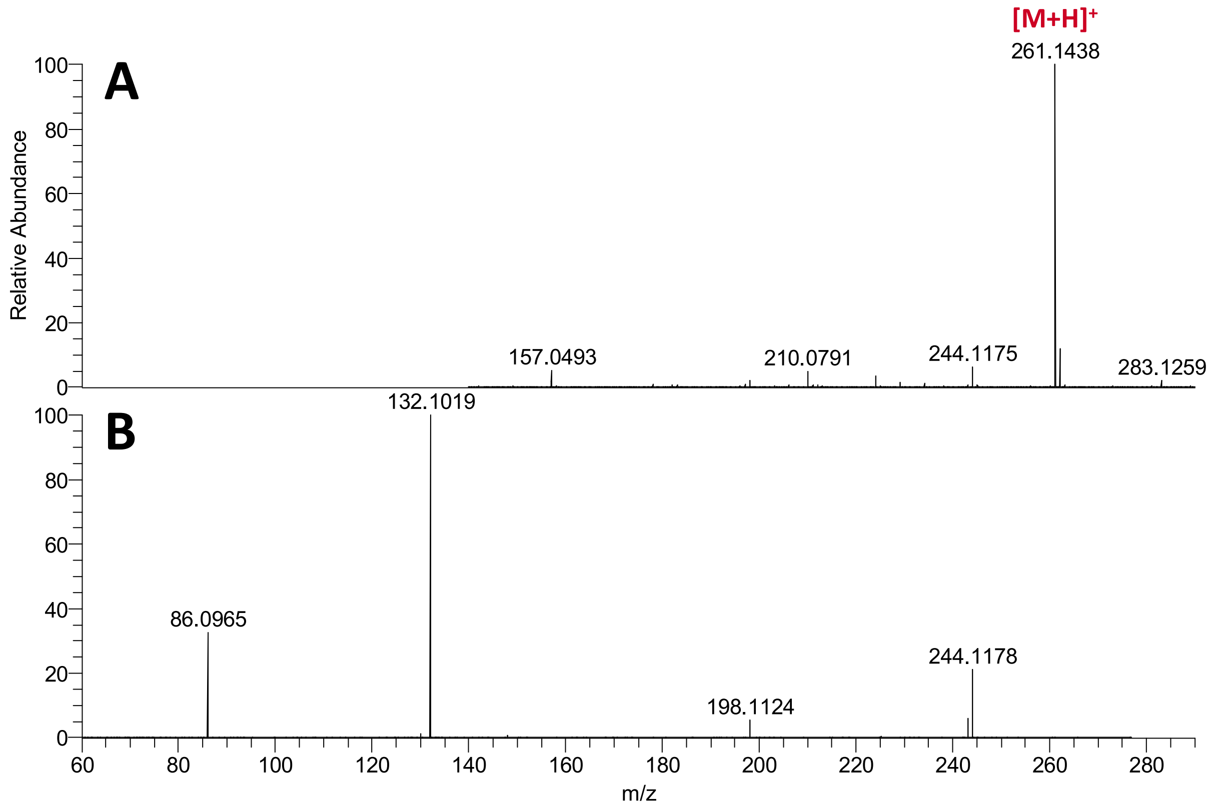

Figure S1B. High resolution ESI mass spectrum of gaburedin B (A) and high resolution MS/MS spectrum of its $[M+H]^+$ ion at *m/z* 261.1438 (B). Reference MS/MS data can be found in https://doi.org/10.1039/C3SC52536H.

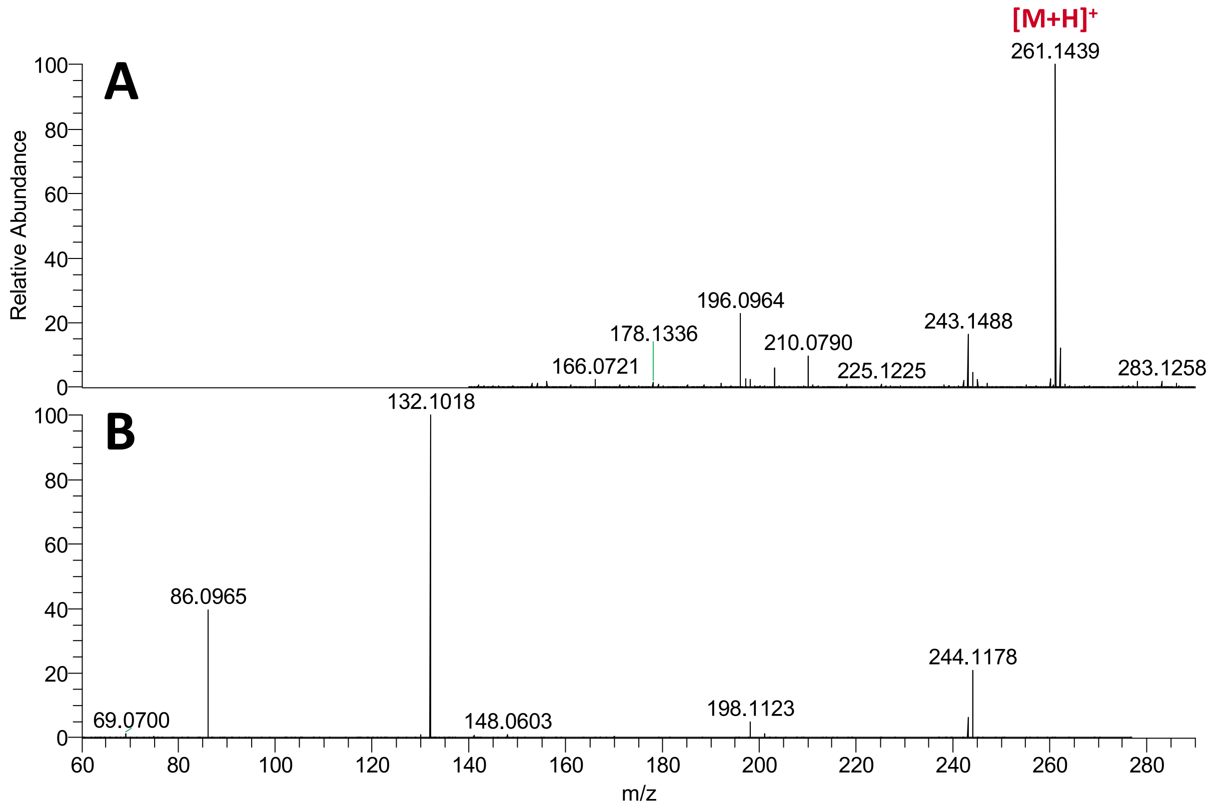

Figure S1C. High resolution ESI mass spectrum of gaburedin C (A) and high resolution MS/MS spectrum of its $[M+H]^+$ ion at *m/z* 261.1439 (B). Reference MS/MS data can be found in https://doi.org/10.1039/C3SC52536H.

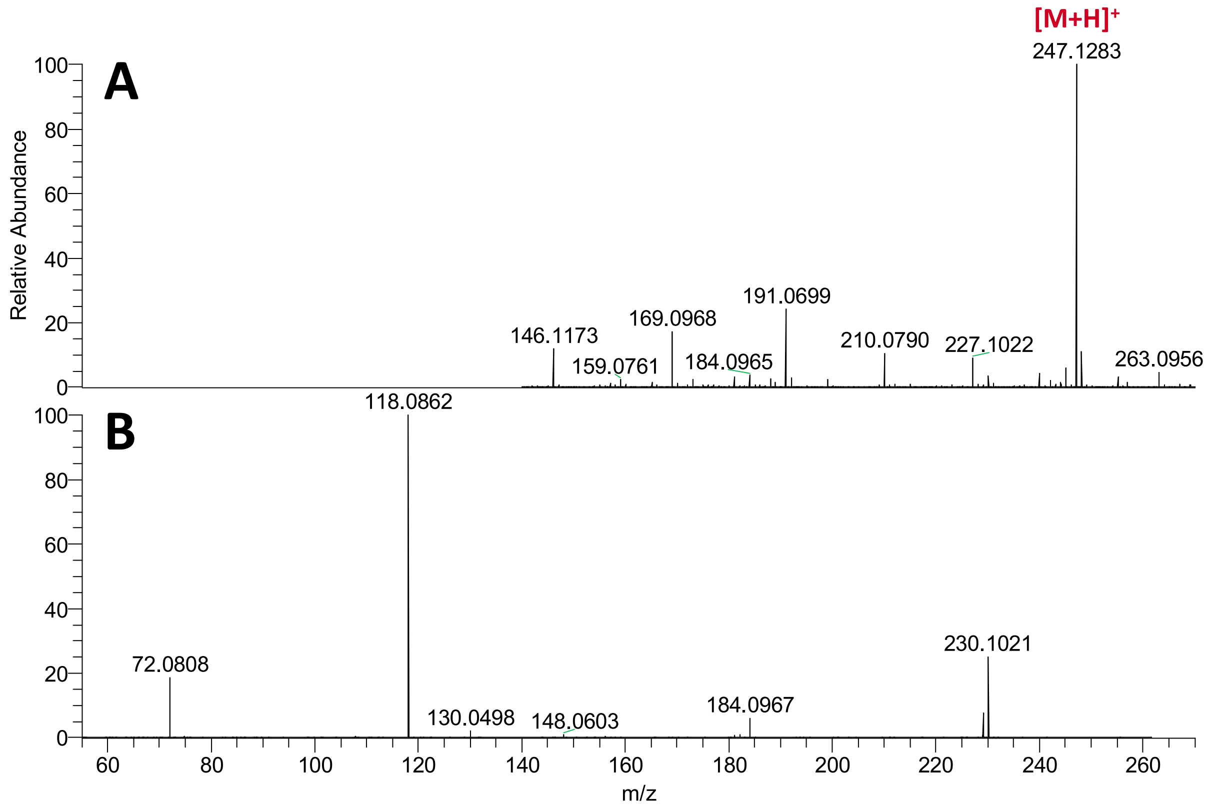

Figure S1D. High resolution ESI mass spectrum of gaburedin D (A) and high resolution MS/MS spectrum of its $[M+H]^+$ ion at *m/z* 247.1283 (B). Reference MS/MS data can be found in https://doi.org/10.1039/C3SC52536H.

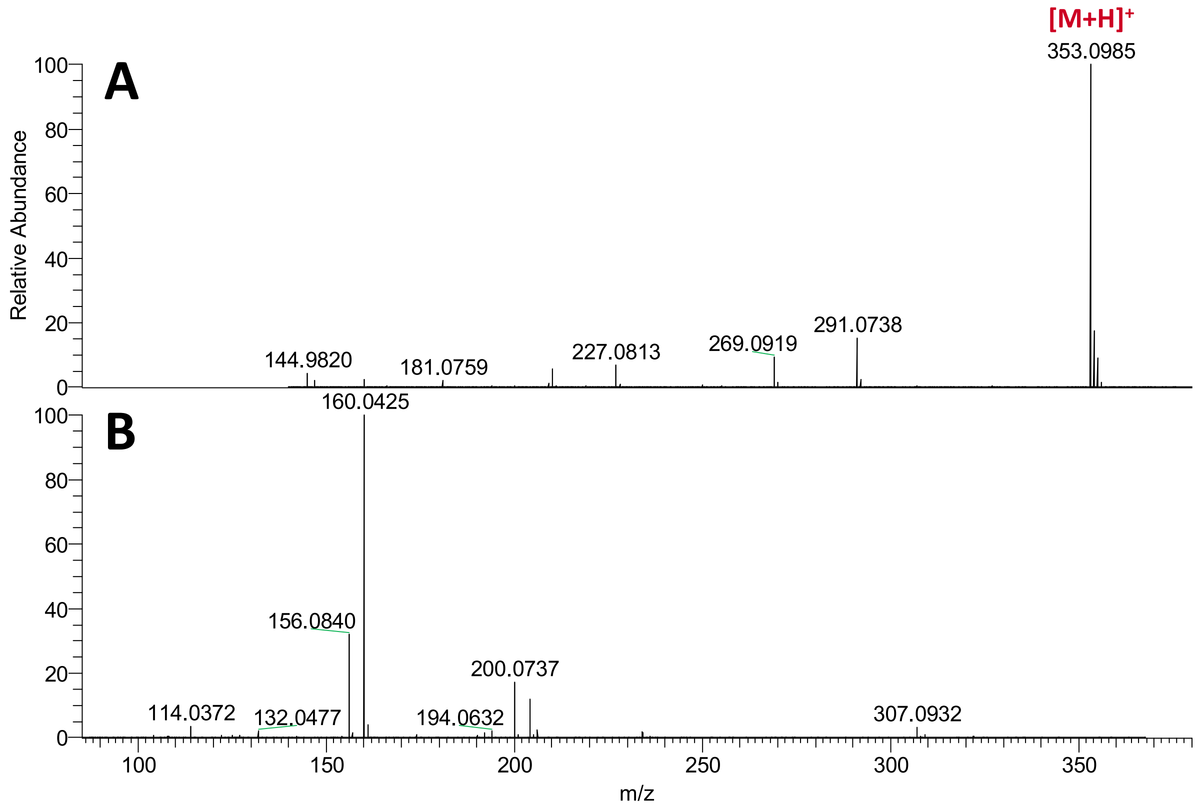

Figure S2A. High resolution ESI mass spectrum of watasemycin A or B (A) and high resolution MS/MS spectrum of its $[M+H]^+$ ion at *m/z* 353.0985 (B). The MS/MS spectra of the two stereoisomers are indistinguishable and only one them is shown. Reference MS/MS data can be found in https://doi.org/10.1039/c6sc03533g.

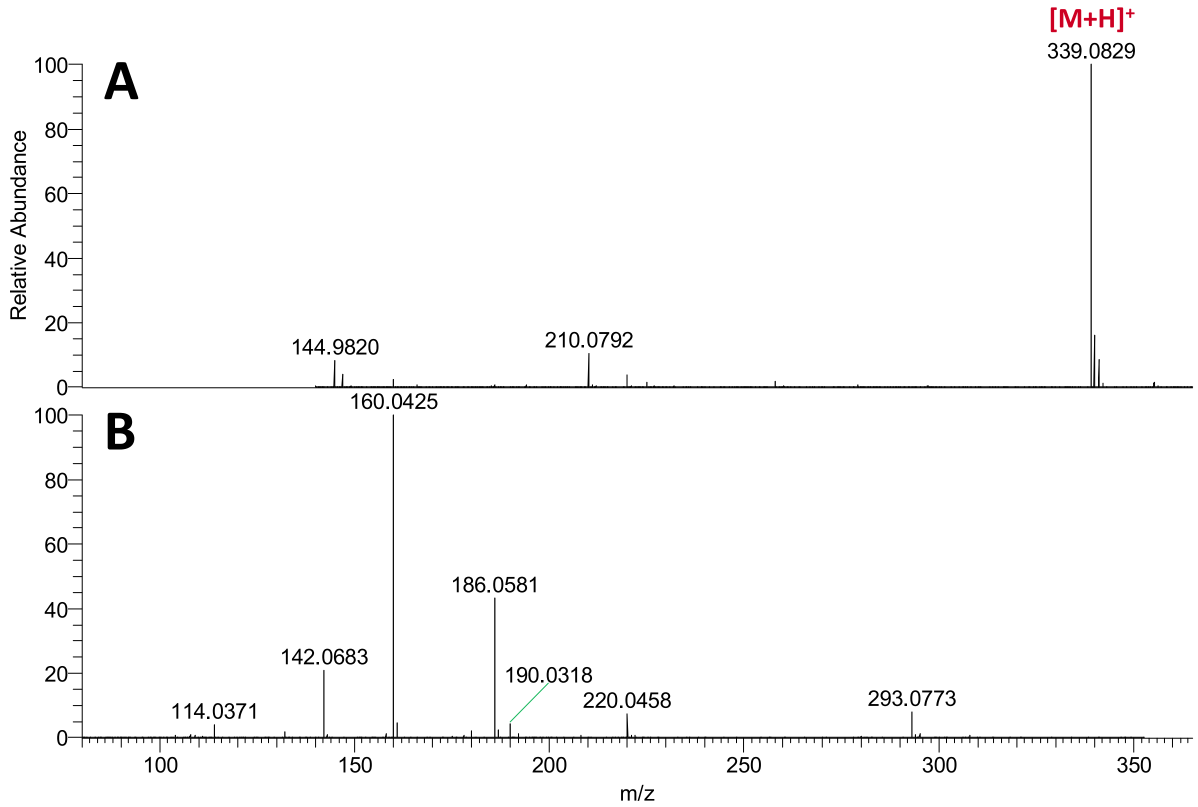

Figure S2B. High resolution ESI mass spectrum of thiazostatin A or B (A) and high resolution MS/MS spectrum of its $[M+H]^+$ ion at *m/z* 339.0829 (B). The MS/MS spectra of the two stereoisomers are indistinguishable and only one them is shown. Reference MS/MS data can be found in https://doi.org/10.1039/c6sc03533g.

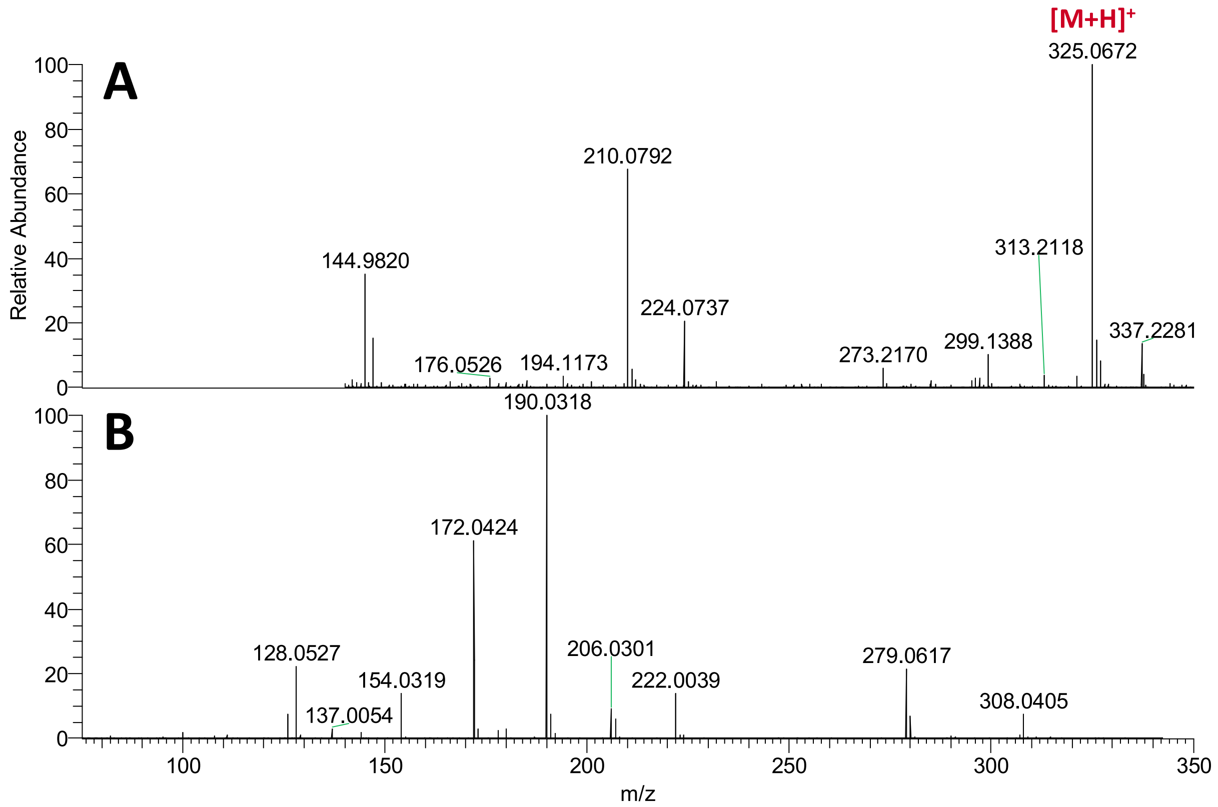

Figure S2C. High resolution ESI mass spectrum of isopyochelin (A) and high resolution MS/MS spectrum of its $[M+H]^+$ ion at *m/z* 325.0672 (B). The MS/MS spectra of the two stereoisomers (see https://doi.org/10.1039/c6sc03533g) are indistinguishable and only one them is shown.

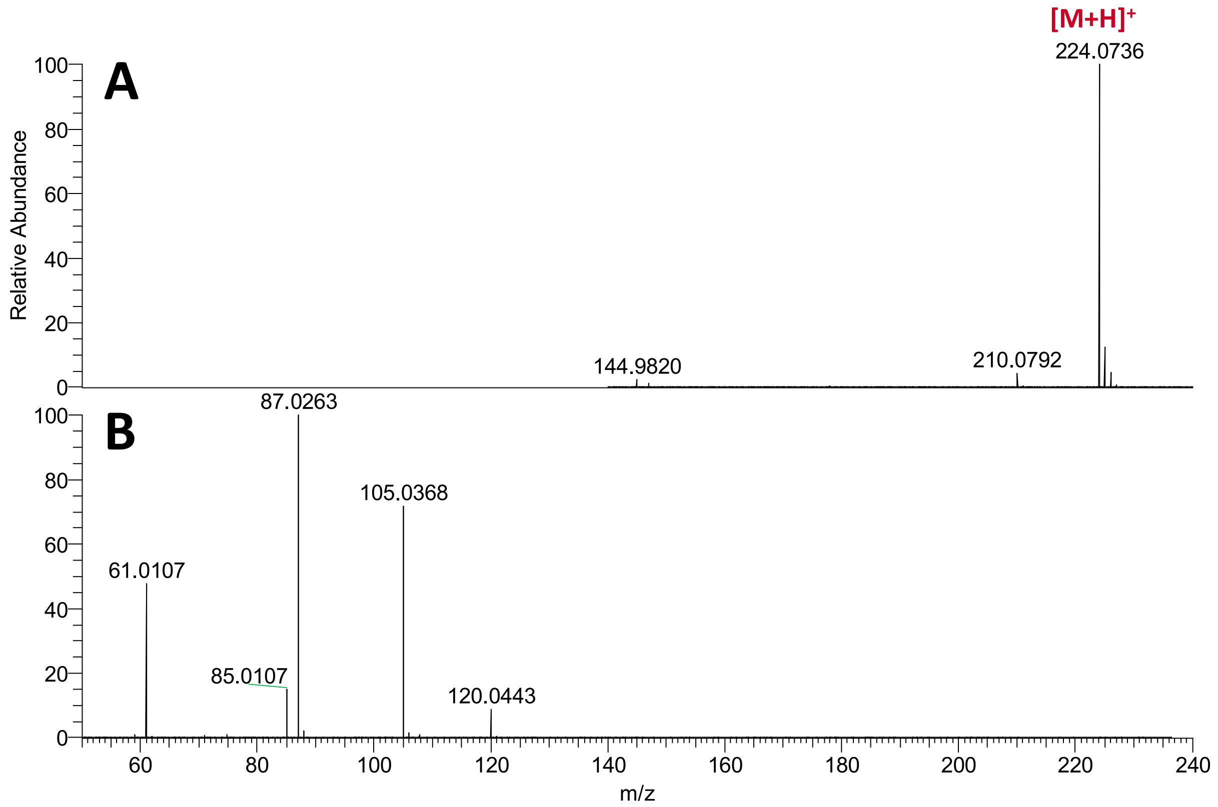

Figure S2E. High resolution ESI mass spectrum of pulicatin A or B (A) and high resolution MS/MS spectrum of its $[M+H]^+$ ion at *m/z* 224.0736 (B). The MS/MS spectra of the two stereoisomers are similar and only one them is shown.

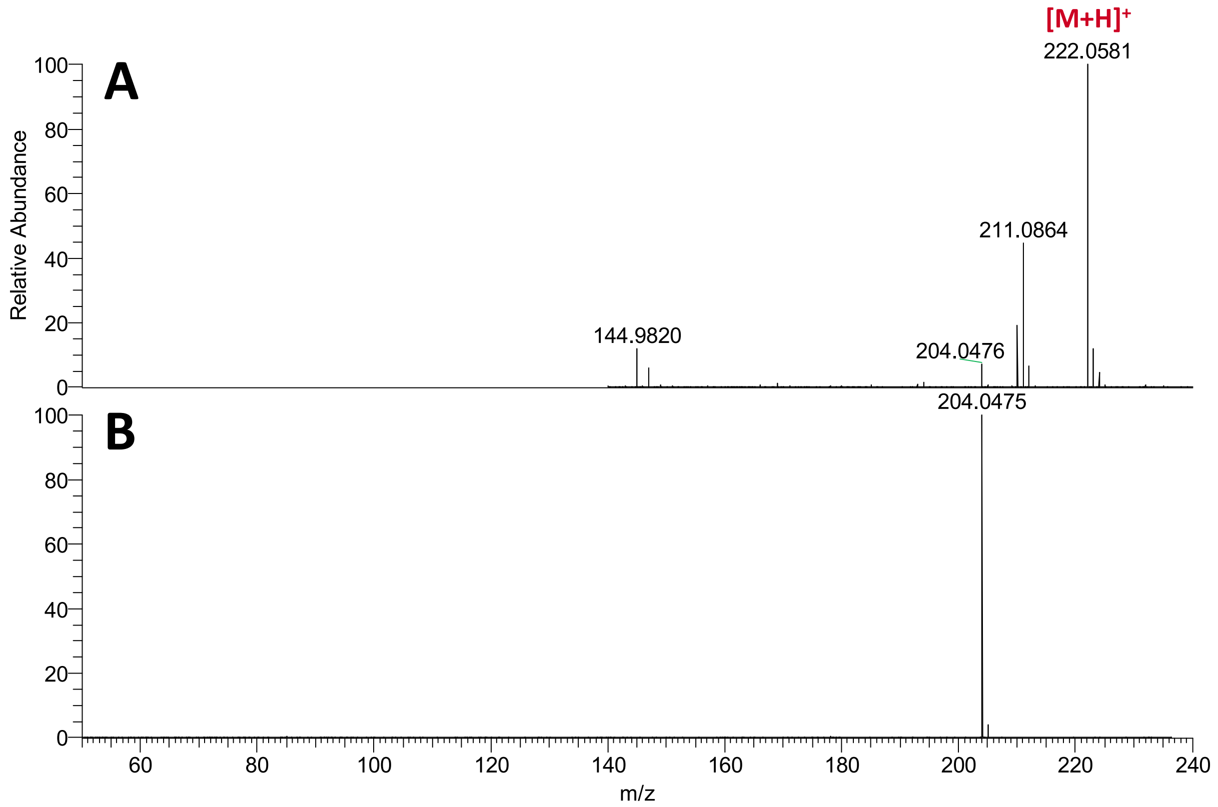

Figure S2F High resolution ESI mass spectrum of pulicatin C (A) and high resolution MS/MS spectrum of its $[M+H]^+$ ion at *m/z* 222.0581 (B).

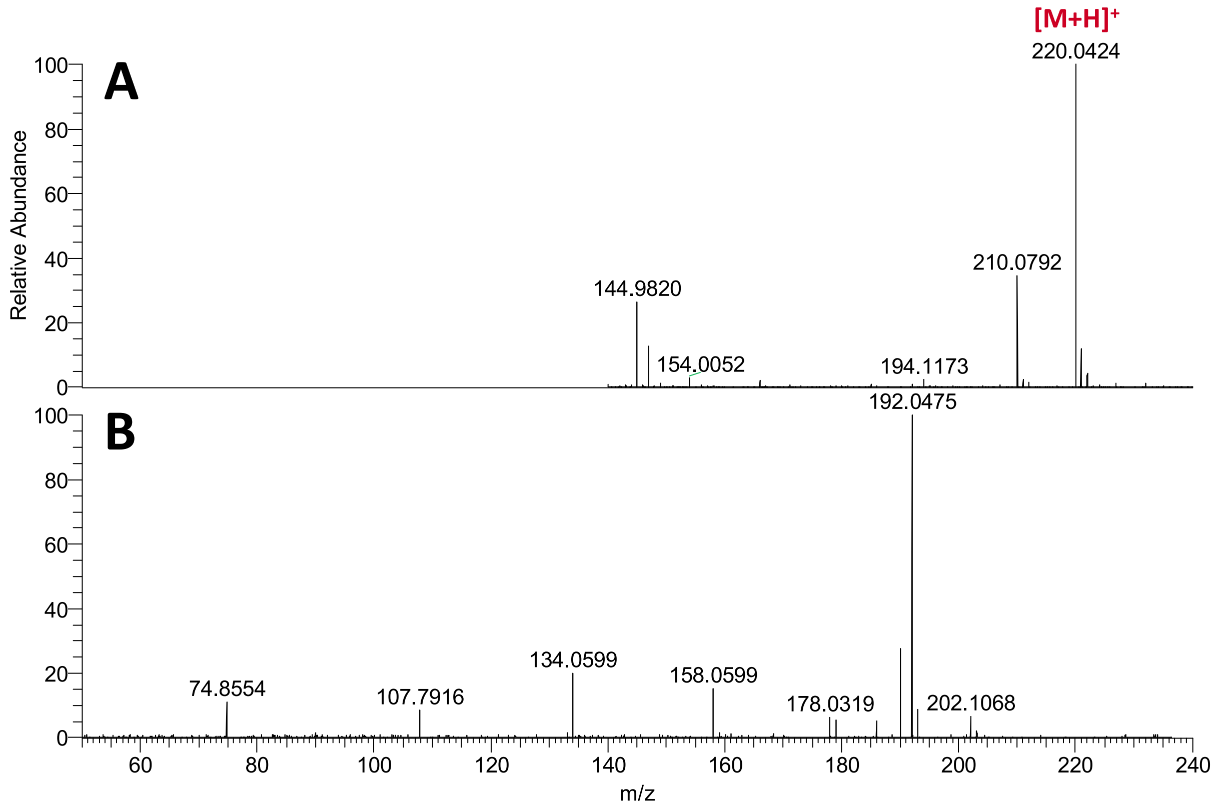

Figure S2G. High resolution ESI mass spectrum of pulicatin D (A) and high resolution MS/MS spectrum of its $[M+H]^+$ ion at *m/z* 220.0424 (B).

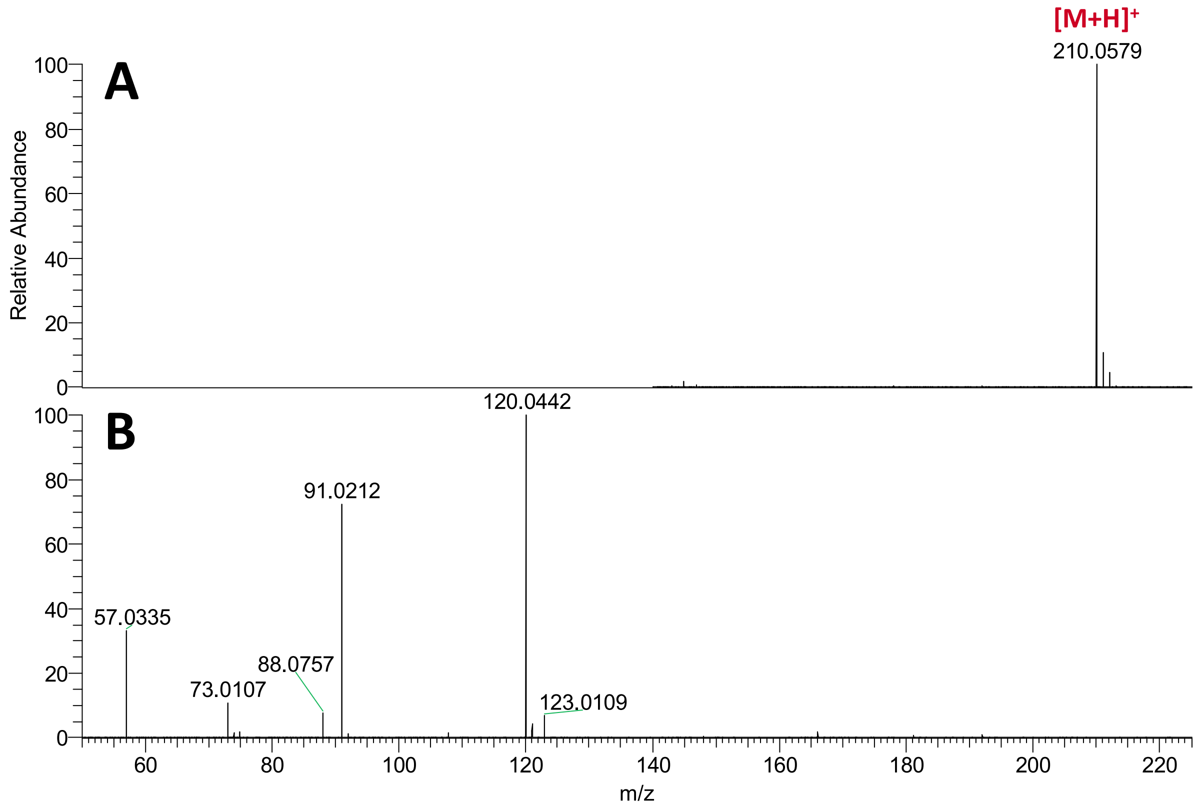

Figure S2H. High resolution ESI mass spectrum of aerugine (A) and high resolution MS/MS spectrum of its $[M+H]^+$ ion at *m/z* 210.0579 (B).

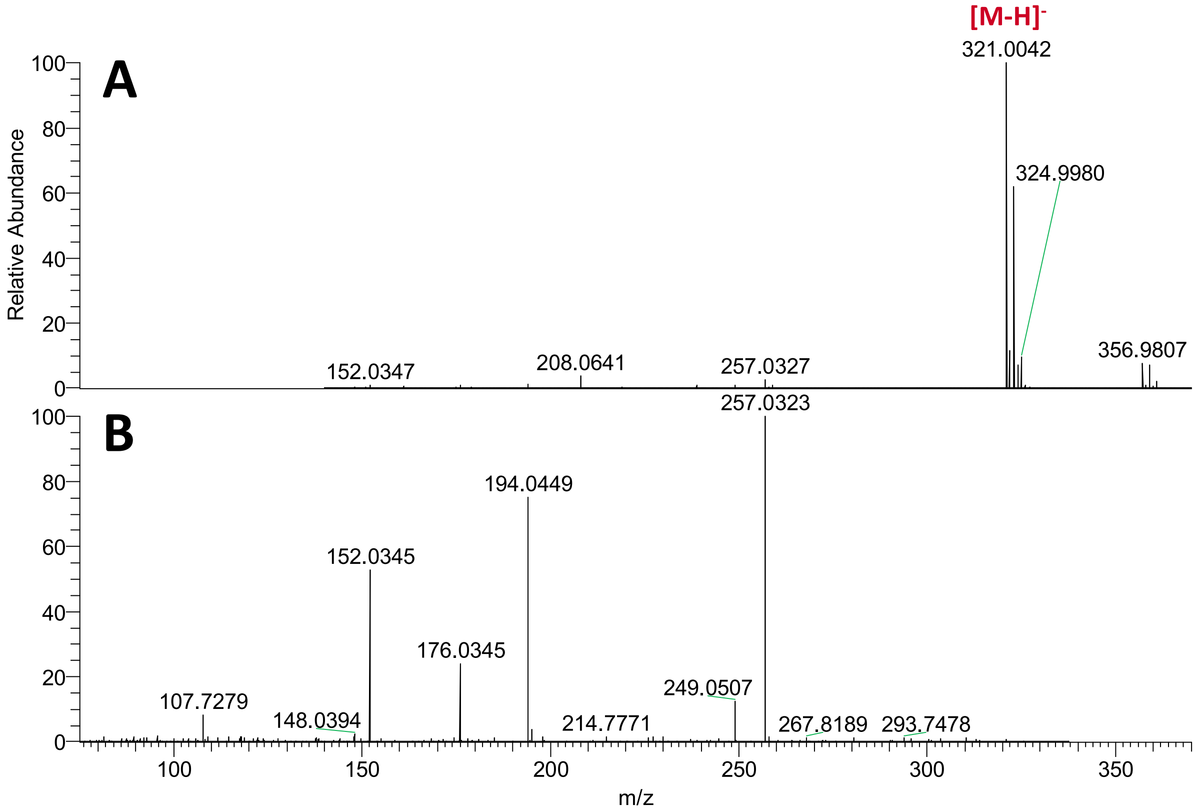

Figure S3. High resolution ESI mass spectrum of chloramphenicol (A) and high resolution MS/MS spectrum of its [M-H]$^-$ ion at *m/z* 321.0042 (B). Reference MS/MS data can be found at https://www.mzcloud.org/DataViewer#Creference1635.

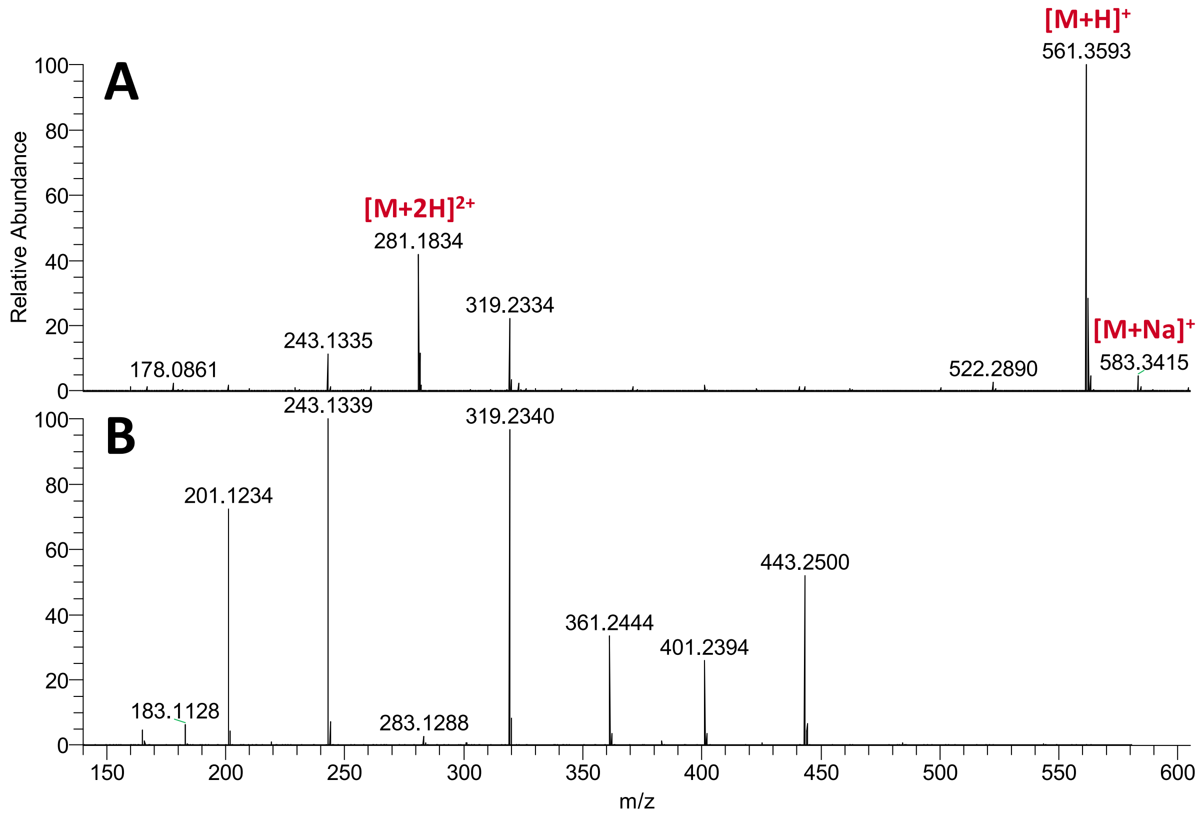

Figure S4. High resolution ESI mass spectrum of desferrioxamine B (A) and high resolution MS/MS spectrum of its $[M+H]^+$ ion at *m/z* 561.3593 (B). Reference MS/MS data can be found in https://doi.org/10.1002/rcm.2295.

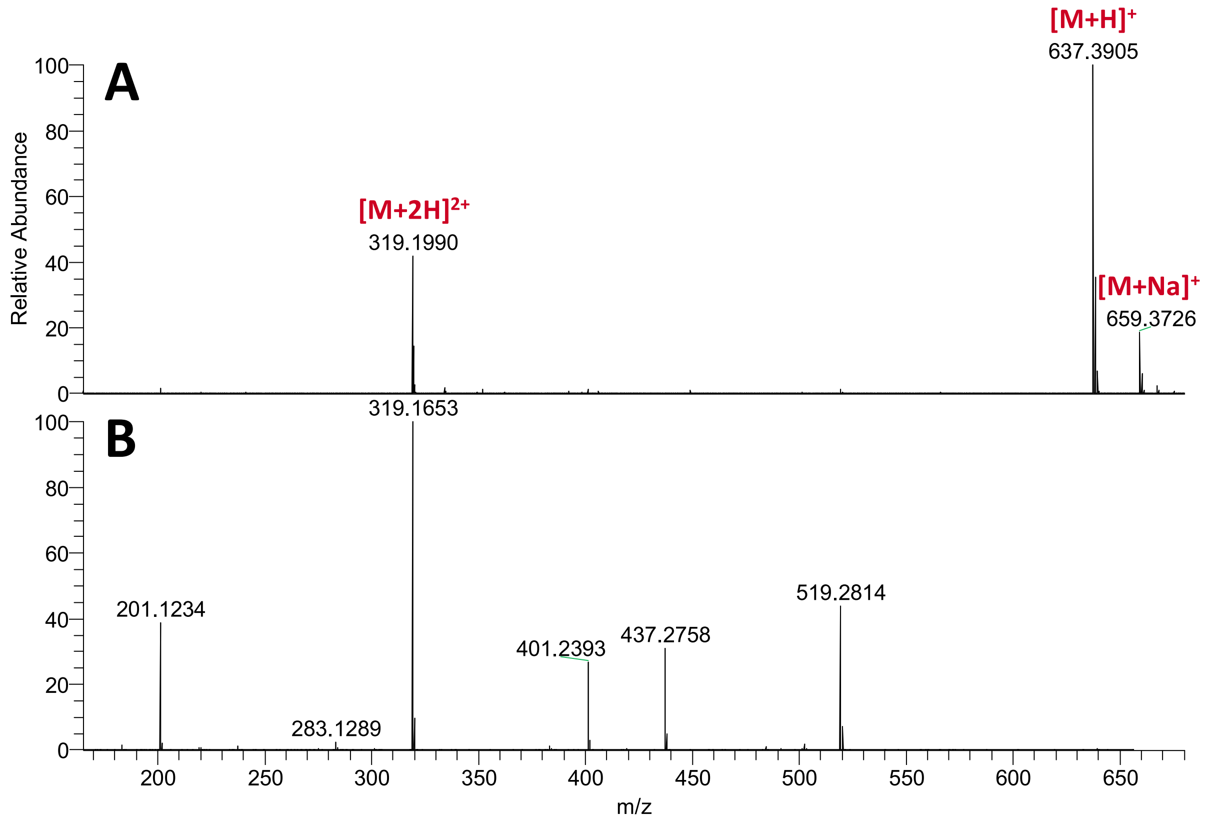

Figure S5. High resolution ESI mass spectrum of legonoxamine A (A) and high resolution MS/MS spectrum of its $[M+H]^+$ ion at *m/z* 637.3905 (B). Reference MS/MS data can be found in https://doi.org/10.1021/ac202623g and https://doi.org/10.1016/j.tetlet.2018.11.063.

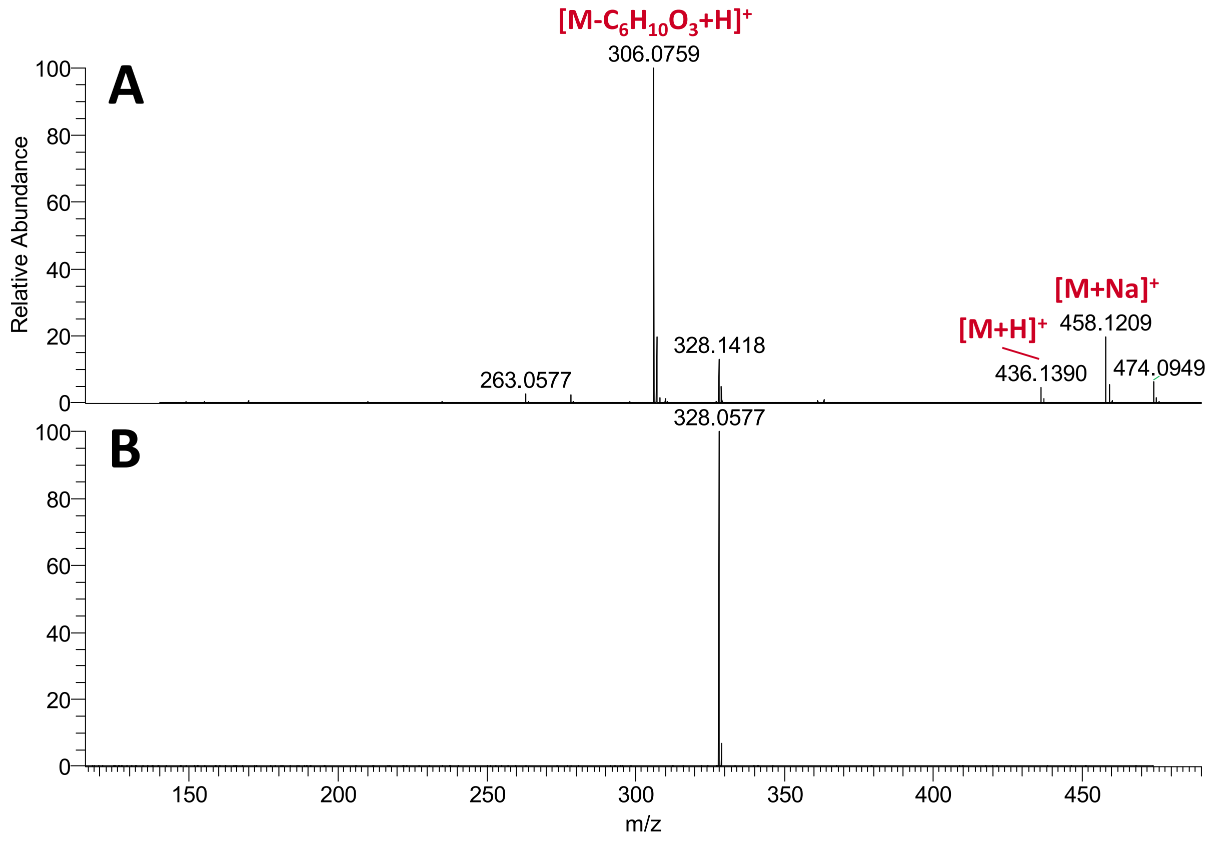

Figure S6A. High resolution ESI mass spectrum of L-digitoxosyl-phenanthroviridin (A) and high resolution MS/MS spectrum of its $[M+Na]^+$ ion at *m/z* 458.1209 (B). Reference MS/MS data can be found in https://doi.org/10.1021/acs.jnatprod.5b00277.

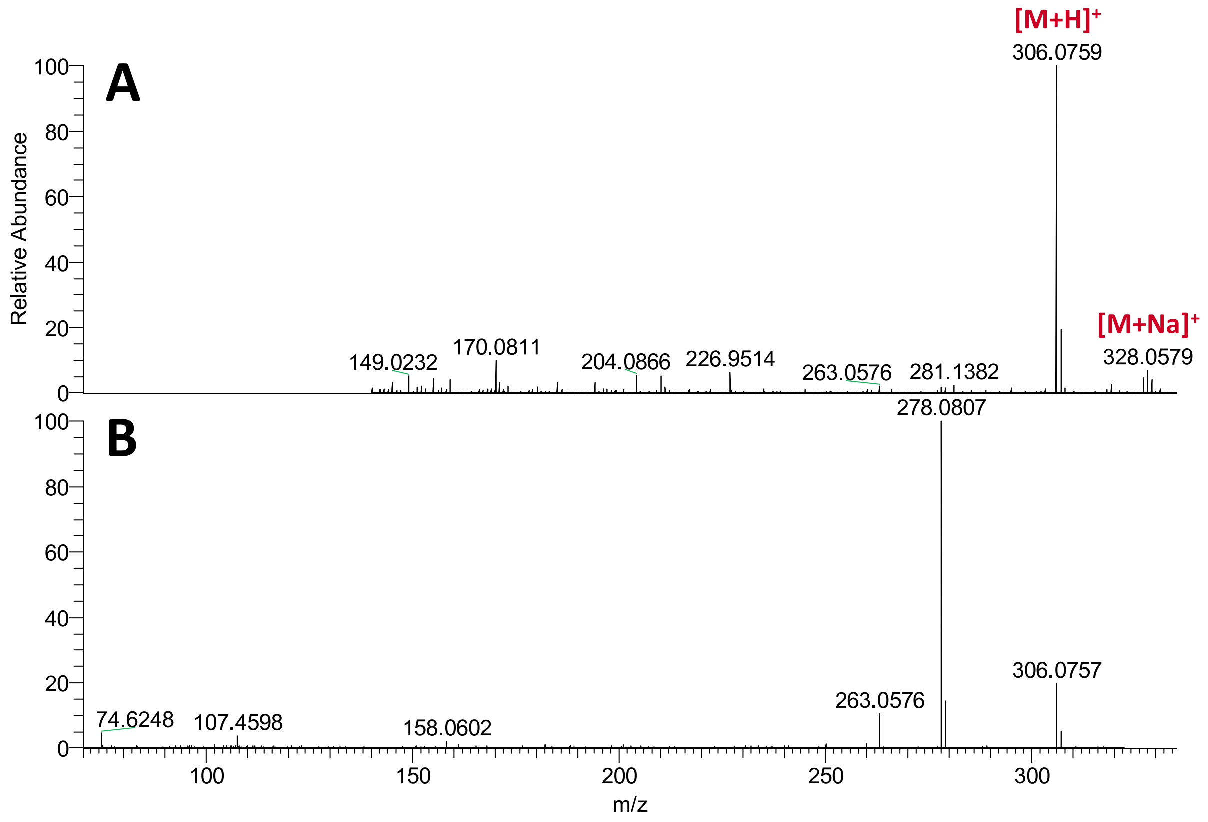

Figure S6B. High resolution ESI mass spectrum of phenanthroviridin aglycone (A) and high resolution MS/MS spectrum of its $[M+H]^+$ ion at *m/z* 306.0759 (B).

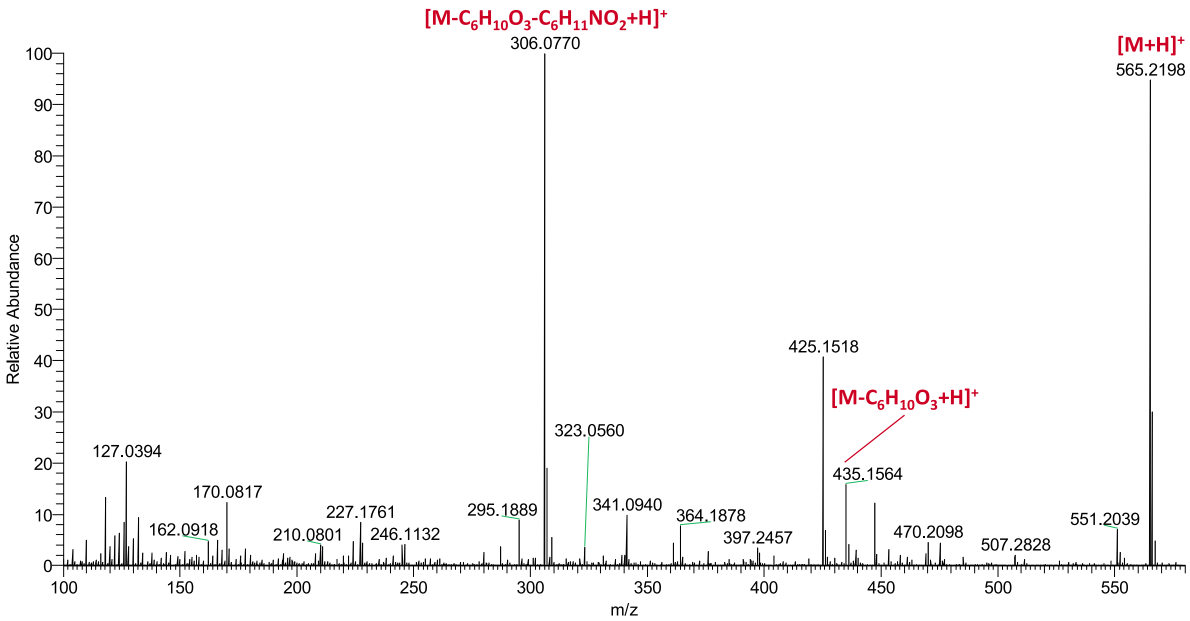

Figure S6C. High resolution ESI mass spectrum of putative jadomycin L-lysine.

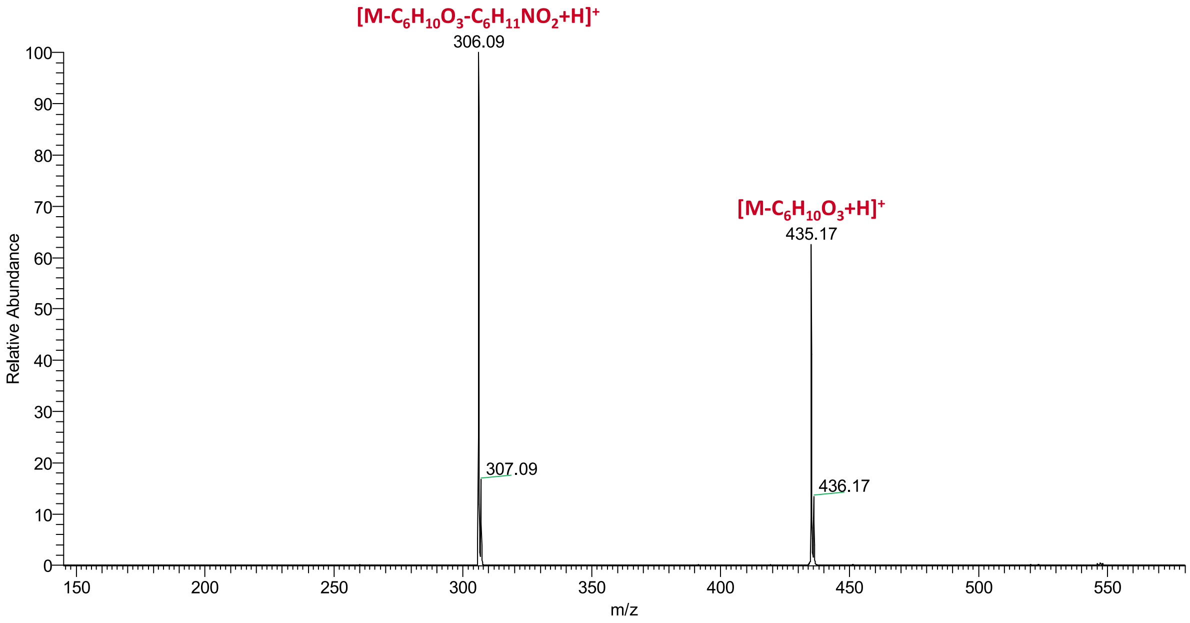

Figure S6D. Low resolution ESI MS/MS spectrum of the $[M+H]^+$ ion of the putative jadomycin L-lysine at *m/z* 565.2198.

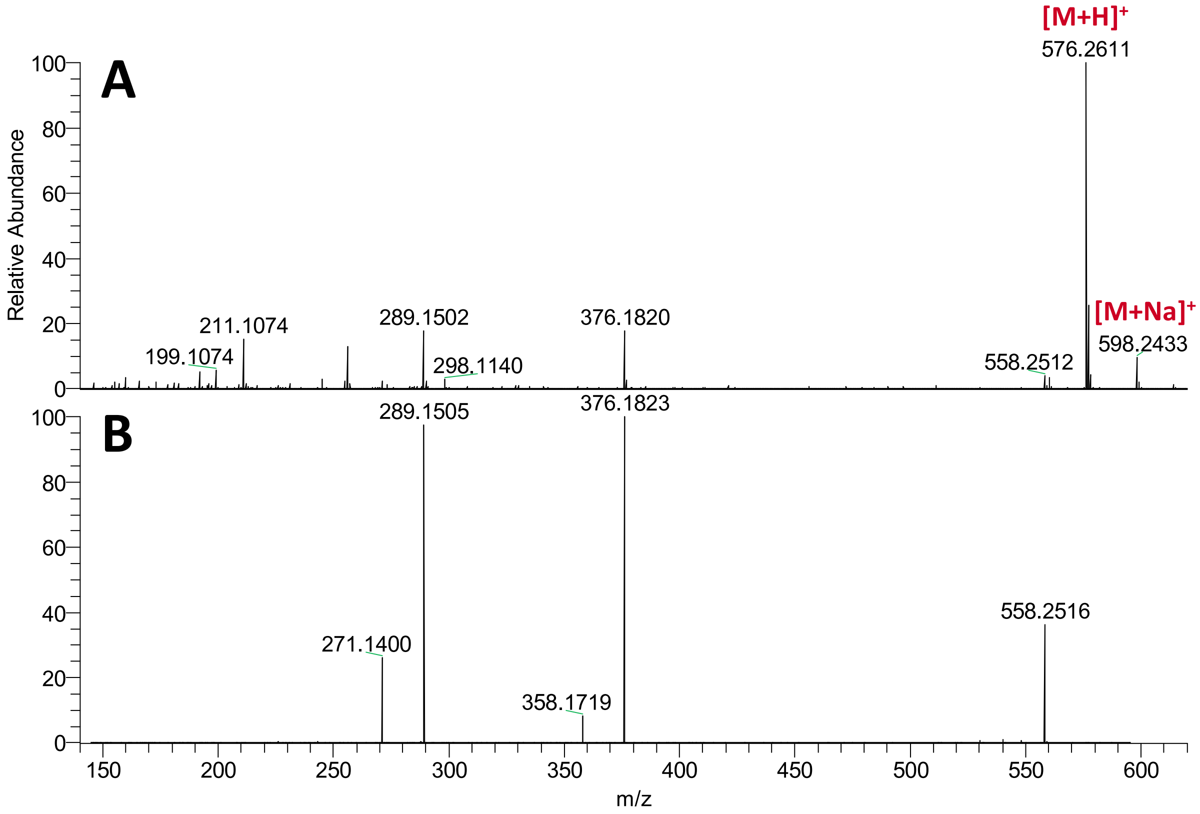

Figure S7. High resolution ESI mass spectrum of foroxymithine (A) and high resolution MS/MS spectrum of its $[M+H]^+$ ion at *m/z* 576.2611 (B). Reference MS/MS data can be found in https://doi.org/10.1021/pr2009115.

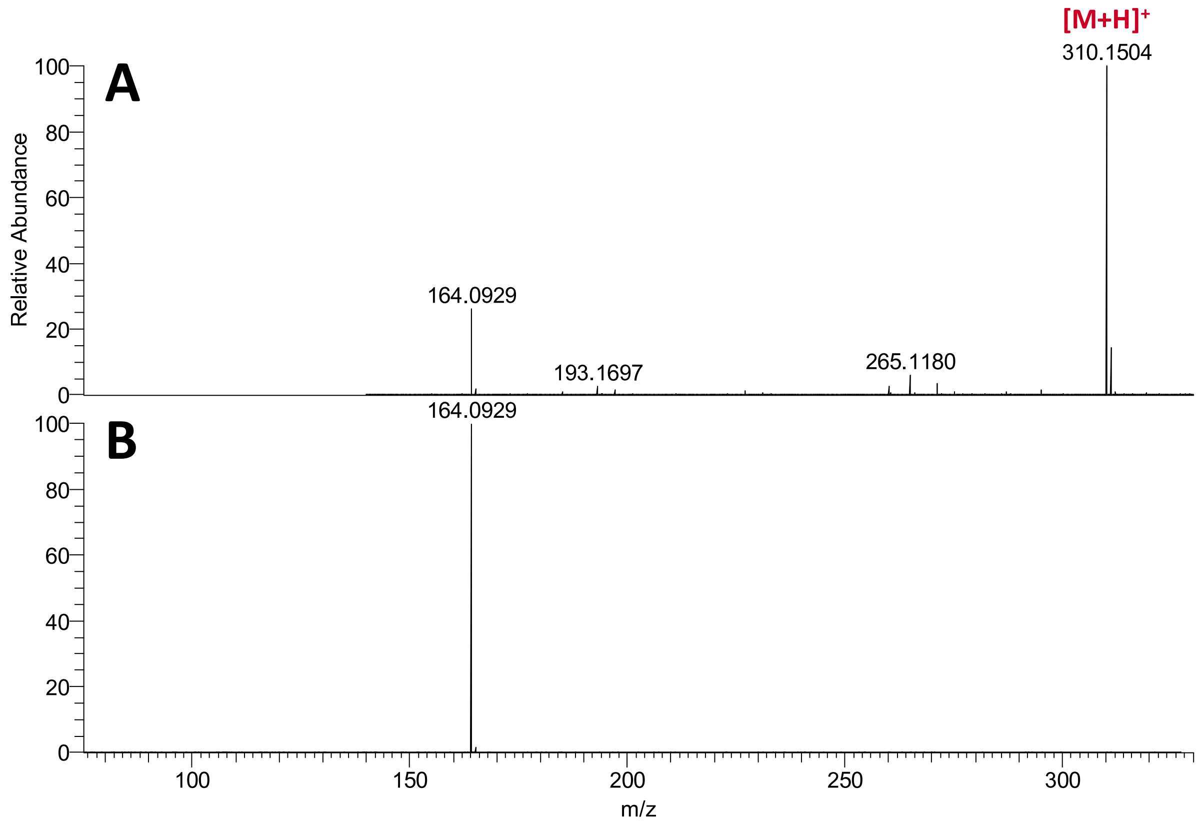

Figure S8A. High resolution ESI mass spectrum of *N*,*N*-dimethyl-*O*-methyladenosine (A) and high resolution MS/MS spectrum of its $[M+H]^+$ ion at *m/z* 310.1504 (B). Reference MS/MS data can be found in https://doi.org/10.1016/S0021-9258(19)50165-X.

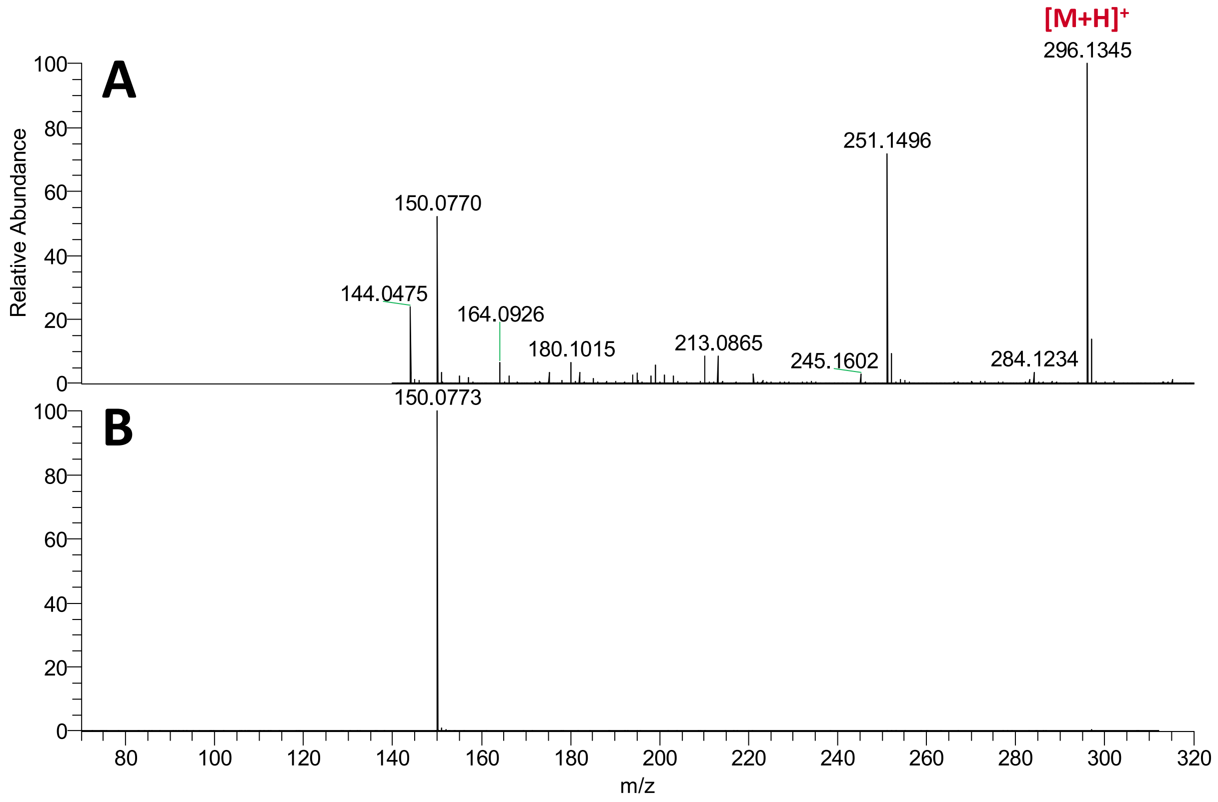

Figure S8B. High resolution ESI mass spectrum of an *N*,*O*-dimethyladenosine isomer (A) and high resolution MS/MS spectrum of its $[M+H]^+$ ion at *m/z* 296.1345 (B). Reference MS/MS data can be found in https://doi.org/10.1016/j.chroma.2020.461181.

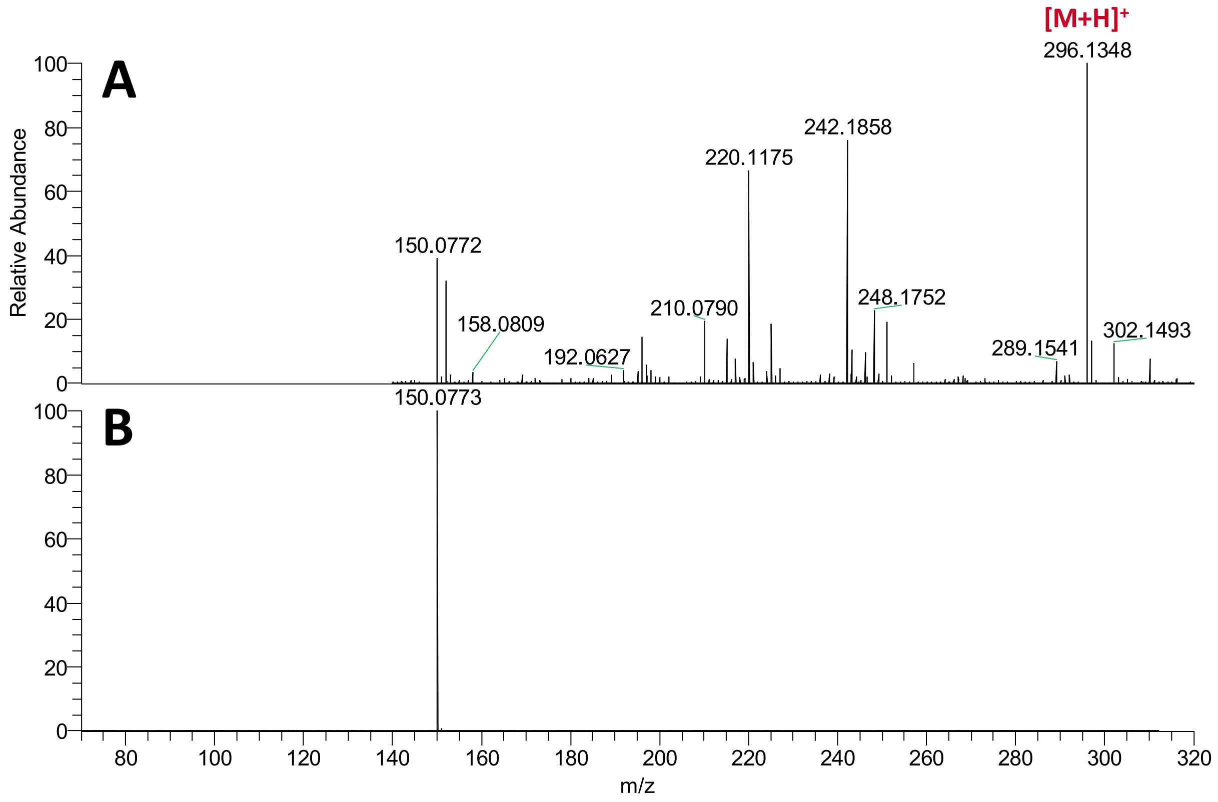

Figure S8C. High resolution ESI mass spectrum of an *N*,*O*-dimethyladenosine isomer (A) and high resolution MS/MS spectrum of its $[M+H]^+$ ion at *m/z* 296.1348 (B). Reference MS/MS data can be found in https://doi.org/10.1016/j.chroma.2020.461181.

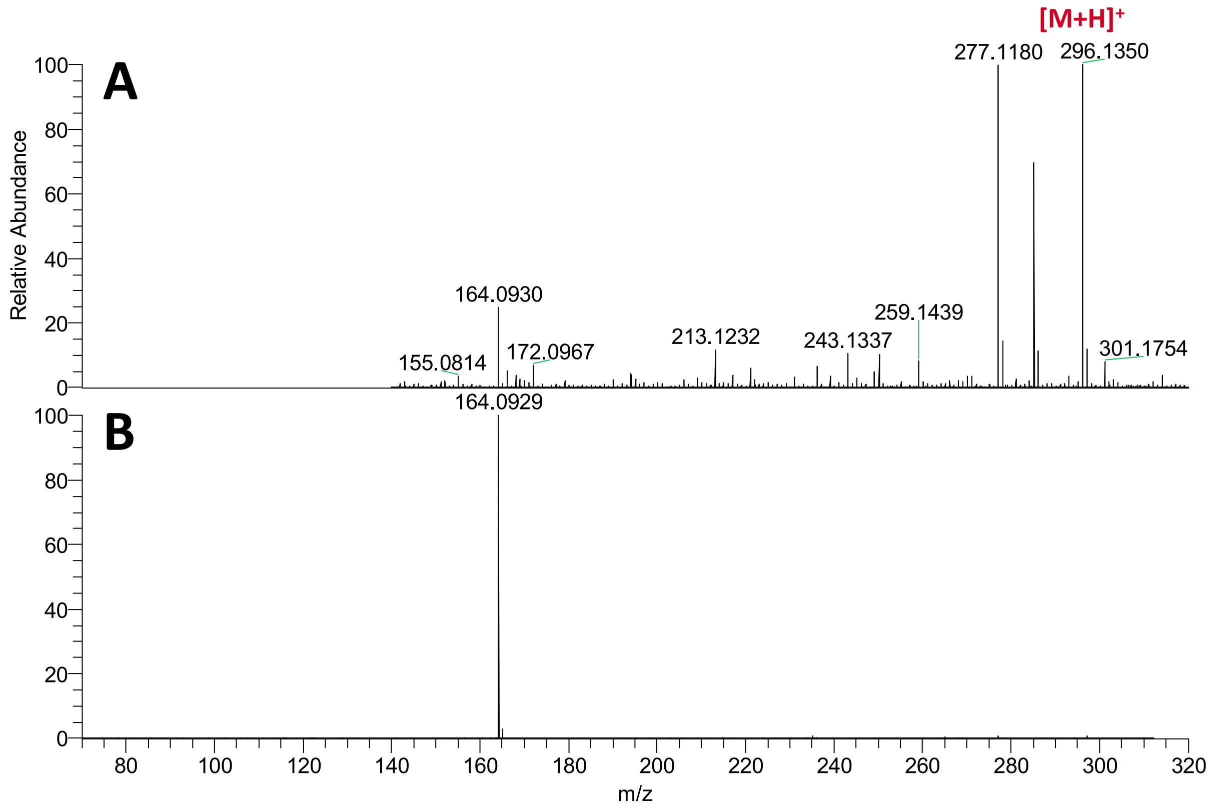

Figure S8D. High resolution ESI mass spectrum of *N*,*N*-dimethyladenosine (A) and high resolution MS/MS spectrum of its $[M+H]^+$ ion at *m/z* 296.1350 (B). Reference MS/MS data can be found in https://doi.org/10.1016/j.chroma.2020.461181.

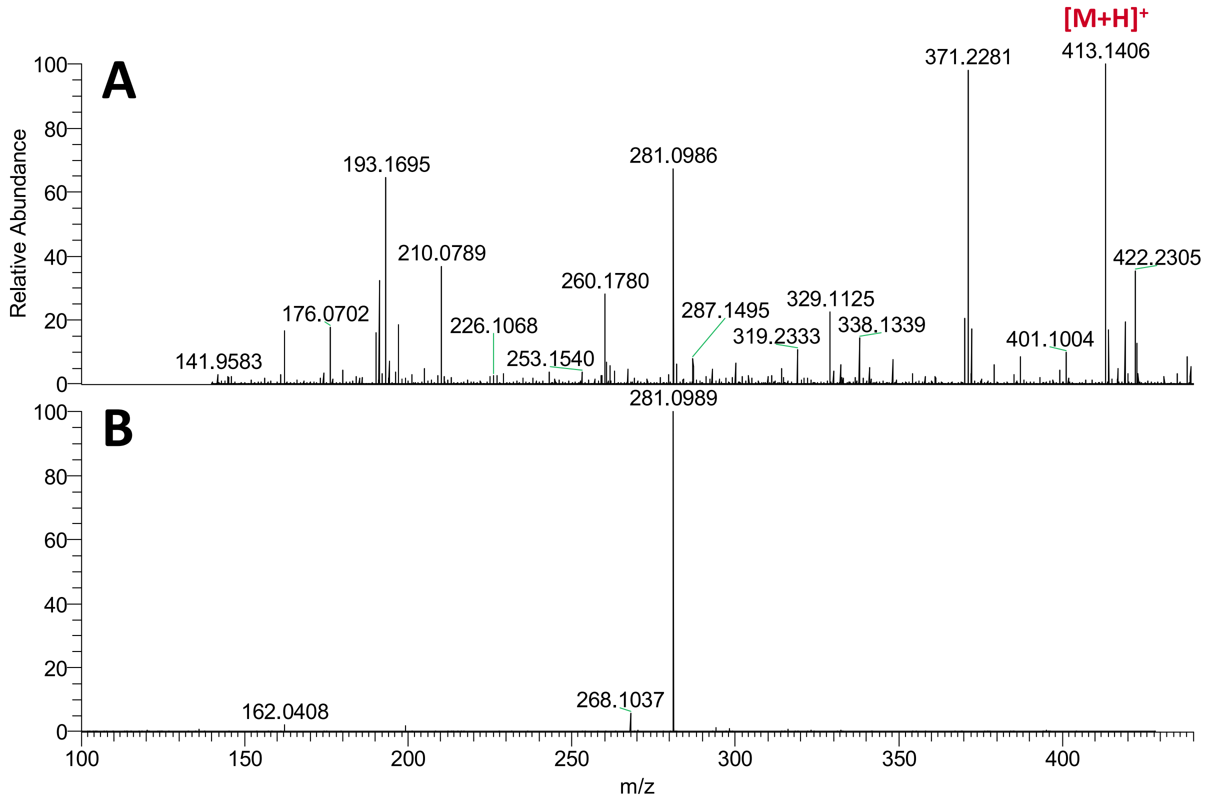

Figure S8E. High resolution ESI mass spectrum of threonylcarbamoyladenosine (A) and high resolution MS/MS spectrum of its $[M+H]^+$ ion at *m/z* 413.1406 (B). Reference MS/MS data can be found in https://doi.org/10.1038/nchembio.1137.

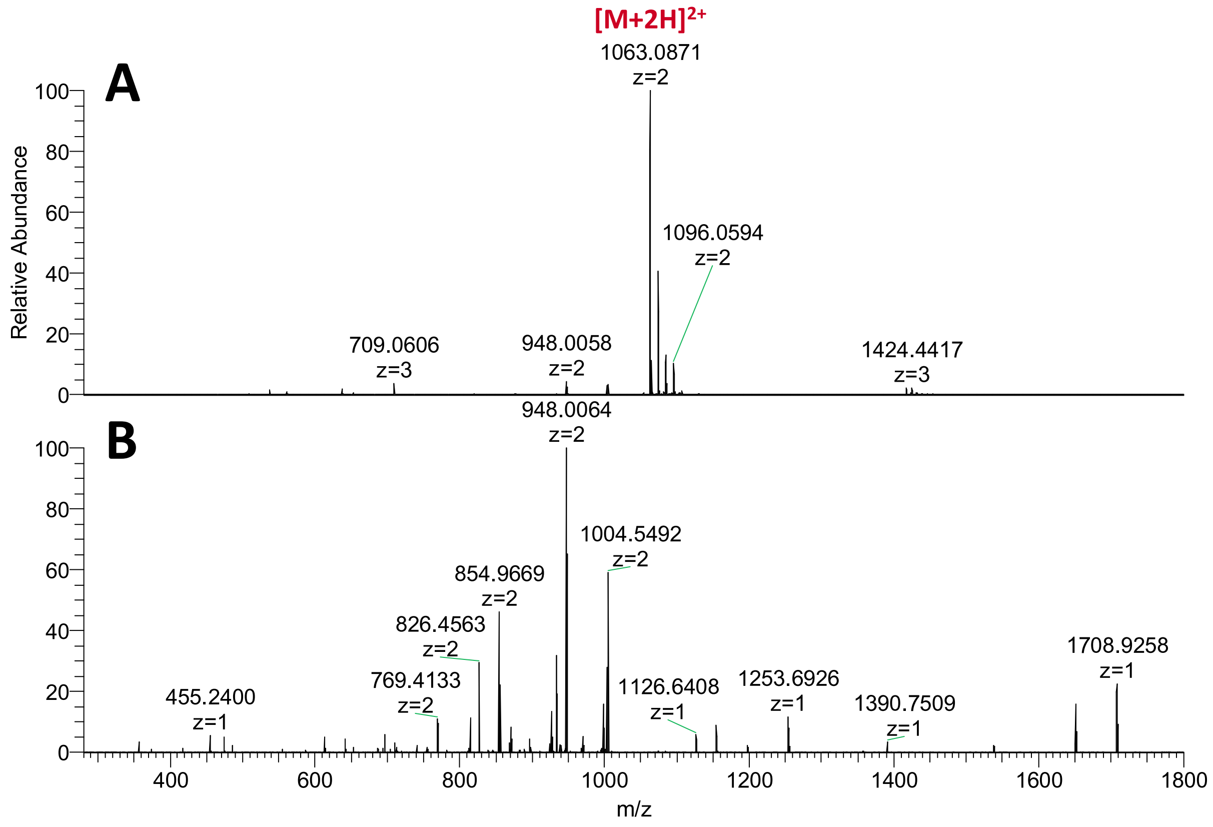

Figure S9. High resolution ESI mass spectrum of PepX (A) and high resolution MS/MS spectrum of its $[M+2H]^{2+}$ ion at *m/z* 1062.5862 (B).

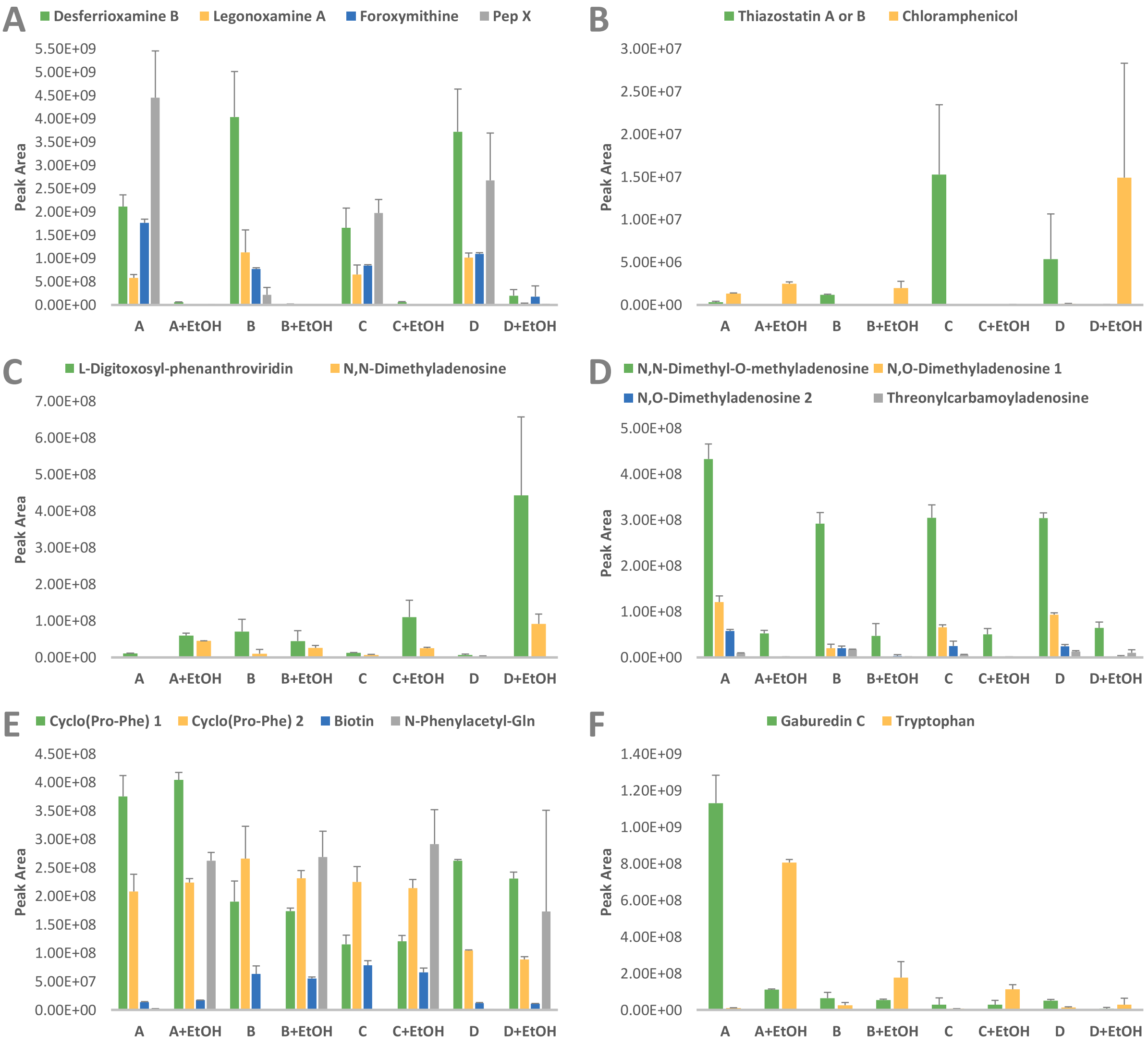

Figure S10. Effect of ethanol shock on the production of secondary metabolites and the relative concentration of other identified compounds in four different batches of MYM cultures of S. *venezuelae* NRRL B-65442 after 48h of cultivation. After 7h of cultivation, either 6% (v/v) of absolute ethanol (+EtOH) or distilled sterile water was added. Each bar represents the average of three replicates (n=3), except for batch "C" (but not "C+EtOH"), where n=2. The error bars represent the standard deviation.

Figure S11.

TCA Cycle

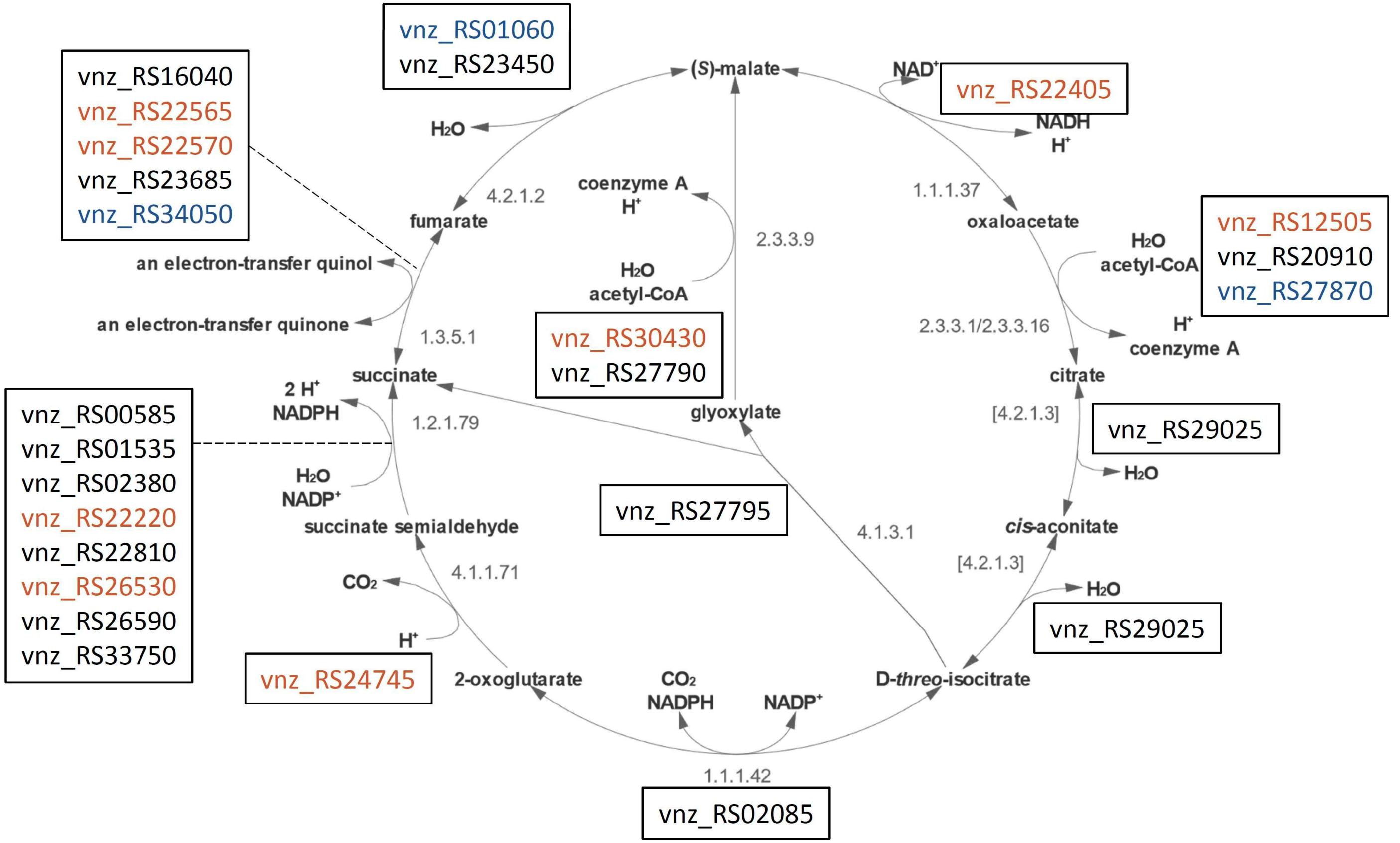

Ref_gene	DEG18h	DEG24h	DEG48h	EC
vnz_RS00585	none	none	none	1.2.1.79
vnz_RS01060	none	down	none	4.2.1.2
vnz_RS01535	none	none	none	1.2.1.79
vnz_RS02085	none	none	none	1.1.1.42
vnz_RS02380	none	none	none	1.2.1.79
vnz_RS12505	up	up	none	2.3.3.-
vnz_RS16040	none	none	none	1.3.5.1
vnz_RS20910	none	none	none	2.3.3.-
vnz_RS22220	up	none	none	1.2.1.79
vnz_RS22405	up	none	none	1.1.1.37
vnz_RS22565	up	none	none	1.3.5.1
vnz_RS22570	up	none	none	1.3.5.1
vnz_RS27790	none	none	none	2.3.3.9
vnz_RS27795	none	none	none	4.1.3.1
vnz_RS22810	none	none	none	1.2.1.79
vnz_RS23450	none	none	none	4.2.1.2
vnz_RS23685	none	none	none	1.3.5.1
vnz_RS24745	up	up	up	4.1.1.71
vnz_RS26530	up	none	none	1.2.1.79
vnz_RS26590	none	none	none	1.2.1.79
vnz_RS27870	none	down	none	2.3.3.-
vnz_RS29025	none	none	none	4.2.1.3
vnz_RS30430	up	up	none	2.3.3.9
vnz_RS33750	None	none	none	1.2.1.79
vnz_RS34050	none	down	none	1.3.5.1
